# Faster implicit motor sequence learning of new sequences compatible in terms of movement transitions

**DOI:** 10.1038/s41539-025-00296-4

**Published:** 2025-01-16

**Authors:** Susanne Dyck, Christian Klaes

**Affiliations:** 1 Department of Neurotechnology, Medical Faculty, Ruhr-University Bochum, Universitaetsstrasse 150, Bochum, 44801 Germany; 2 International Graduate School of Neuroscience, Ruhr-University Bochum, Universitaetsstrasse 150, Bochum, 44801 Germany; 3 Neurosurgery, University hospital Knappschaftskrankenhaus Bochum, In der Schornau 23-25, Bochum, 44892 Germany

**Keywords:** Consolidation, Human behaviour, Learning and memory

## Abstract

New information that is compatible with pre-existing knowledge can be learned faster. Such schema memory effect has been reported in declarative memory and in explicit motor sequence learning (MSL). Here, we investigated if sequences of key presses that were compatible to previously trained ones, could be learned faster in an implicit MSL task. Participants trained a motor sequence before switching to a completely new sequence, to a compatible sequence with high overlap in ordinal positions, or to an incompatible sequence with low overlap, while the compatible and incompatible sequences had the same overlap in movement transitions. We observed accelerated learning in the Compatible and Incompatible groups compared to the New group, if participants trained for 3 sessions before switching to the altered sequence. Overall, our study suggests facilitative learning of implicit motor sequences that are compatible in movement transitions, if the previous sequence has been trained extensively.

## Introduction

When we learn something new, we do not always have to start from zero. Relying on our previous knowledge, we can learn new information faster, if it is consistent or compatible with what we already know^1^. Hereby, the compatible information is integrated into a pre-existing schema, an associative map that represents knowledge abstracted from previous experiences^1^. Experimentally, schema memory was reported in rats that learned flavor-location associations^2,3^, and in humans in the declarative memory domain, for instance in an associative learning task^4^.

In the motor memory domain, schema memory might also play an important role in integrating new movements into previously learned skills. For example, after training a sequence of dance steps, you could face a situation where you have to change or add one step to the choreography. Learning sequential actions is ubiquitous in everyday life and corresponds to motor sequence learning (MSL). When learning a motor sequence, two processes are involved: On the one hand, you acquire knowledge about the individual elements of the sequence and their temporal order, which constitutes the explicit process. It is characterized by the subjects’ awareness that learning has taken place, conscious recollection, reportability, and highly flexible usage^5–8^. On the other hand, you need to bind the individual elements of the sequence into a single, skilled behavior through motor practice^9^. This particular process is implicit. Here, performance improves without the intention to learn, the subject is unaware of the learning process, and the implicit sequence memory is typically indirectly tested via performance on a task^5,10^. Besides the level of awareness, the explicit and implicit components also differ in their temporal dynamics: A fast and a slow component could be extracted that contribute to MSL, corresponding to the explicit and implicit process, respectively^11,12^. Typically, the early stage of motor learning is dominated by explicit processes, while implicit processes play a role in later stages, i.e. in the automatization of the learned skill^13^.

In the lab environment, MSL is often investigated using the Serial Reaction Time Task (SRTT)^14^, a forced-choice reaction time task where participants perform button presses cued by spatially congruent visual stimuli. The stimuli follow a fixed repeating sequence, interspersed by trials consisting of random sequences. The reaction time (RT) typically decreases over the course of training. Since the RT reduction is higher in the sequence condition compared to the random condition, it is attributed to sequence-specific motor learning. The random condition is included to capture general practice effects, and confounding factors such as fatigue and motivation^15,16^. The sequence-specific performance improvements are even evident in implicit versions of the SRTT, where participants are not instructed about the presence of an underlying sequence and learning is incidental rather than intentional^14^.

When associating the concept of schema memory and MSL, one evident question is how compatibility is defined for motor sequences. Let’s take the example of learning the PIN “4, 2, 1, 6”. You acquire knowledge about individual elements plus their ordinal positions, e.g. knowing that “4” is the first number, “2” is the second number, etc. Furthermore, you learn movement transitions from one element to the next, e.g. knowing that “2” is following “4”, etc. Thus, when learning a new PIN, for example, “4, 2, 1, 5”, the overlap in ordinal positions (3/4) would be high, while only 2 of the 3 previous movement transitions remained from the original PIN. However, the new PIN “2, 1, 6, 4” would have no overlap in ordinal positions (0/4), while also preserving 2 of the 3 previous movement transitions. Whether one of those two new PINs would be easier to learn than the other, depends on what the nature of compatibility is for motor sequences and on the nature of the memory representation (i.e. have you explicitly learned that the second number in your PIN is 2 or are you relying on the movement transition between the individual key presses when remembering your PIN).

Recently, King et al.^17^ have extended the concept of schema memory to the motor memory domain. In an explicit MSL task (a bimanual explicit version of the SRTT^14^), participants trained a motor sequence for one session. The following day they performed a second training session where they, depending on their experimental group, continued to train the same sequence (Same group), trained a non-overlapping new sequence (New group), a sequence that had a high overlap in ordinal positions (Compatible group) or a sequence with a low overlap in ordinal positions (Incompatible group) compared to the previously trained sequence in session 1. Importantly, the compatible and incompatible sequences were designed such that they shared the same movement transitions (50% learned movement transitions shared with the previously trained sequence and 50 % novel movement transitions), to investigate if the overlap in ordinal positions or movement transitions was an important factor in learning new motor sequences. King et al.^17^ showed that for explicit MSL, new sequences can be learned faster via rapid integration of the new motor information if they overlap with a previously learned motor sequence in terms of ordinal positions. They suggest that during training, a cognitive-motor schema that binds motor events to their ordinal positions is developed, with the involvement of hippocampal-cortical networks^17^. Since the hippocampus has been reported in both explicit and implicit MSL^18,19^, the question arises if a schema memory-like effect is also evident in implicit MSL. This question also relates to transfer learning and generalization, which both play an important role in skill learning. While the implicit learning system is thought to be more inflexible than the explicit learning system^20–22^, at least a partial transfer has been reported to a new context where the motor or perceptual information was altered^22^. Moreover, implicit learning is assumed to take place on a slower timescale than explicit learning^12,23^. It has been proposed that motor sequence learning involves a fast, effector-independent component and a slower effector-dependent component, representing visuo-spatial and motor information, respectively^24^.

Thus, the aim of this study was to investigate (i) if there is a facilitative learning effect for compatible sequences in implicit MSL; and, in case of a facilitative learning effect (ii) the basis of compatibility for implicit sequences (ordinal positions or movement transitions); and (iii) whether the facilitative learning effect of compatible sequences in implicit MSL depends on the amount of previous training.

We employed a similar task design as in the study by King et al.^17^, albeit using an implicit version of the bimanual SRTT^14^ without instructing participants about the presence of a repeating sequence. Following their sophisticated study design using the four experimental groups (Same, Compatible, Incompatible, New)^17^, allowed us to test whether compatibility in ordinal positions or movement transitions affected learning a new motor sequence in implicit MSL. To study the effect of previous training, we performed 2 experiments varying in the number of training sessions: In experiment 1 (4 days), participants trained the motor sequence for 3 training sessions before changing to the compatible/incompatible/new or continuing the same sequence in session 4; in experiment 2 (2 days), participants trained the motor sequence for one training session before performing the compatible/incompatible/new sequence.

## Results

### Experiment 1 (4 days)—Sequence-specific learning across training sessions

The primary measurement of learning in the SRTT^14^ and thus, in our bimanual version of the SRTT, are the reaction times (RTs). A reduction of RTs reflects improved performance in terms of speed. Another performance measure is accuracy, as in the percentage of correct button presses. Moreover, we combined these two behavioral performance measures into a compound measure by scaling the RT of correct key presses with the accuracy in that particular block (see Methods for details).

**Fig. 1:**
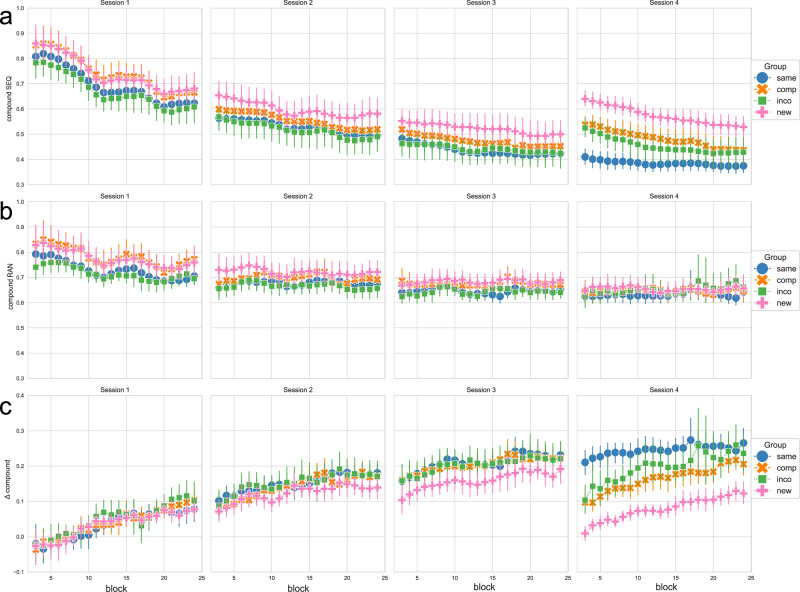
Performance measures in experiment 1 across the 4 training sessions and experimental groups, in the sequence condition, the random condition, and the contrast between both. **a** shows the compound measure, which is based on the RT of correct key presses scaled by the accuracy/number of errors, thus combining RT and accuracy, in the sequence condition (SEQ); **b** shows the compound measure as a combination of RT and accuracy in the random condition (RAN); **c** shows the Δ compound measure, which is obtained by contrasting the random and sequence condition, to obtain a measurement of the performance that is specific to sequence-learning. The experimental groups “same", “comp" (= compatible), “inco" (= incompatible), and “new" are color-coded in blue, orange, green, and pink, respectively. Each column shows a training session. Data is depicted using a sliding window approach (moving average smoothing, *n* = 3). Vertical bars represent the 95 % confidence intervals.

Figure 1 a) shows the compound measure for the implicit sequence condition over the course of training sessions, for each experimental group. A corresponding figure showing the RTs and the accuracy across training sessions and experimental groups can be found in the Supplementary material (Supplementary Fig. 1).

As a control condition that captures general practice effects that are not specific to the performance of the implicit motor sequence, participants also executed pseudo-random sequences in each block. Figure 1b shows the compound measure for the random condition across training sessions and groups. The corresponding RTs and the accuracy in the random condition can be found in the Supplementary material (Supplementary Fig. 2).

To derive performance gains specific to sequence learning, we contrasted the compound measures from the sequence and random control conditions by subtracting the compound measure of the implicit sequence condition from the compound measure of the random condition. Hence, filtering out improvements in performance resulting from general practice effects (see also Pollok et al.^25^ and Dyck and Klaes^26^). Figure 1c shows the obtained Δ compound values as a proxy for performance changes specific to sequence learning, for each experimental group, throughout blocks and training sessions. Here, values above 0 represent better performance in the sequence versus the random condition, thus indicating motor sequence learning (MSL).

**Fig. 2:**
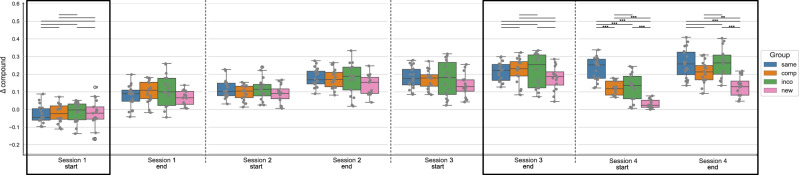
Sequence-learning specific performance measures, represented by Δ compound, at the start and end of each training session, in experiment 1 (4 days). The Δ compound was obtained by contrasting the random and implicit conditions for a sequence-specific learning measure. The first/last 4 blocks of a session are included for the start/end of the session, respectively. The experimental groups “same", “comp" (= compatible), “inco" (= incompatible), and “new" are color-coded in blue, orange, green, and pink, respectively. The data is depicted in box plots or box-and-whisker plots. The box shows the lower and upper quartile of the data, representing 50 % of the compound scores, while the whiskers extend to show the rest of the distribution. Grey dots show the Δ compound of individual subjects in the experimental group and at the specific time points. The black boxes mark the specified timepoints of interest: start of training the initial motor sequence - session 1 start; end of training the initial motor sequence - session 3 end; and session 4 where a new/compatible/incompatible or the same sequence has been trained. At those timepoints, statistical comparisons of the Δ compound measures between the experimental groups were performed (black vertical lines), using paired t-tests, with * representing *p* ≤ 0.05, ** *p* ≤ 0.01 and *** representing *p* ≤ 0.001 (Bonferroni corrected for multiple comparisons).

In the next step, for statistical analysis, we split the data into different timepoints during the training sessions, i.e. the start and the end of each training session, by taking the mean of the first or last 4 blocks of each session, respectively. Figure 2 shows the Δ compound values at the start and end of each training session per experimental group. A mixed ANOVA with the factor timepoint (within-subject) and group (between-subject) revealed a significant effect of the factor timepoint (*F* = 129.568, *p* < 0.001), a significant effect of the factor group (*F* = 5.442, *p* = 0.002) and a significant interaction of timepoint and group (*F* = 5.256, *p* < 0.001) on the Δ compound values. Moreover, for each experimental group, repeated measures ANOVA revealed a significant effect of the factor timepoint on the Δ compound values (*p* < 0.001 for all groups). Post hoc tests showed that the performance in each experimental group significantly improved from the start of session 1 to the end of session 3 (*p* < 0.001 for all groups). When the new, compatible, or incompatible sequence was introduced at the start of session 4, the performance was significantly reduced compared to the end of session 3 (*p* = 0.002, *p* < 0.001, *p* = 0.001, for the New, Compatible, and Incompatible group, respectively), while the performance in the Same group did not change between the end of session 3 and the start of session 4 (*p* = 1.). The Δ compound values in the New, Compatible, and Incompatible groups significantly increased within session 4, i.e. from the start to the end of session 4 (*p* = 0.02, *p* < 0.001, *p* = 0.04), while there was no performance gain evident in the Same group (*p* = 1.). The results of the ANOVA plus all post-hoc comparisons (per experimental group) can be found in the Supplementary material (Supplementary Tables 1-7).

To compare the performance across the experimental groups, we focused on the behavioral data at four specific time points of interest: at the start of training as a control for the baseline performance; at the end of session 3, representing the end of training of the original motor sequence for all groups (except for the Same group); the start of session 4, to have an estimate of the initial learning phase of the altered sequence; and the end of session 4 to see the effect of online learning for the altered motor sequence within one training session (the areas marked by black borders in Fig. 2). We performed one-way ANOVAs with the between-subject factor group at those specific timepoints, to see if the Δ compound values differed across groups.

At the start of session 1, the factor group had no significant effect on the Δ compound scores (*F* = 0.146, *p* = 0.932). Similarly, at the end of session 3, there was no significant difference in the Δ compound scores between the 4 groups (Welch’s ANOVA: *F* = 1.245, *p* = 0.309). Thus, suggesting that all experimental groups had a comparable baseline performance and reached a similar performance and levels of MSL by the end of session 3, which is expected since up to this time point, all experimental groups practiced the same motor sequence. At the start of session 4, where the implicit sequence was altered based on the experimental group, there was a significant main effect of the factor group on the Δ compound scores (Welch’s ANOVA: *F* = 42.968, *p* < 0.001). Post hoc tests revealed a significant difference in the performance of the New group with all other groups (all *p* < 0.001), i.e. a decreased performance in the New group compared to the other groups. The performance in the Same group was significantly higher compared to all other groups (all *p* < 0.001). Only the Compatible and Incompatible groups showed no difference in performance (*p* = 1.). Thus, the performance significantly deteriorated in the 3 experimental groups where a new/compatible/incompatible sequence was introduced, compared to the group that continued to perform the original motor sequence. However, the performance in the Compatible and Incompatible groups was similar, while the performance in the New group showed a higher impairment. At the end of the last training session, the Δ compound measures still significantly differed between the experimental groups (Welch’s ANOVA: *F* = 12.635, *p* < 0.001). As previously, the comparison between the New and all other groups showed significant differences in Δ compound measures (new and same: *p* < 0.001, new and incompatible: *p* < 0.001, new and compatible: *p* = 0.008), with the New group having the lowest performance across groups. The performance between the Compatible, Incompatible, and Same groups showed no significant difference (compatible and incompatible: *p* = 0.761, compatible and same: *p* = 0.481, incompatible and same: *p* = 1.).

### Experiment 1 (4 days) - Performance in novel versus learned movement transitions

**Fig. 3:**
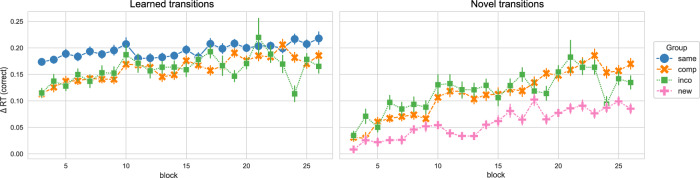
Performance in the learned versus novel movement transitions in experiment 1, over the course of training session 4. The Δ reaction times (RTs) for correctly executed learned and novel movement transitions in experiment 1, across blocks in session 4, separated by experimental groups, are depicted. The Δ RT was obtained by contrasting the random and implicit conditions for a sequence-specific learning measure. While both the Compatible and Incompatible sequences contain 50 % learned and 50 % novel transitions, the Same sequence contains only learned transitions, and the New sequence contains only novel transitions. The experimental groups “same", “comp" (= compatible), “inco" (= incompatible), and “new" are color-coded in blue, orange, green, and pink, respectively. Vertical bars represent the 95 % confidence intervals.

**Fig. 4:**
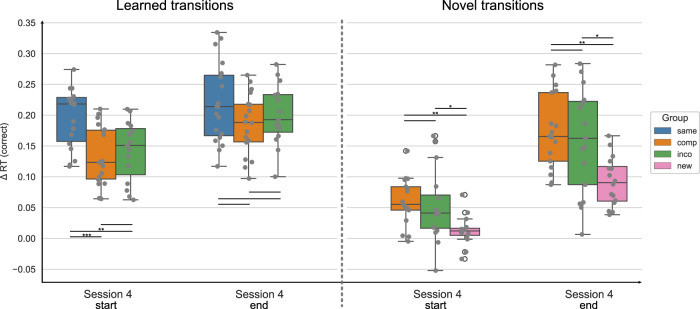
Performance in the learned versus novel movement transitions in experiment 1, session 4. The Δ reaction times (RTs) for correctly executed learned and novel movement transitions are depicted at the start and end of session 4, separated by experimental groups. The Δ RT was obtained by contrasting the random and implicit conditions for a sequence-specific learning measure. The first/last 4 blocks of a session are included for the start/end of the session, respectively. While both the Compatible and Incompatible sequences contain 50 % learned and 50 % novel transitions, the Same sequence contains only learned transitions, and the New sequence contains only novel transitions. The experimental groups “same", “comp" (= compatible), “inco" (= incompatible), and “new" are color-coded in blue, orange, green, and pink, respectively. The data is depicted in box plots or box-and-whisker plots. The box shows the lower and upper quartile of the data, representing 50 % of the RT scores, while the whiskers extend to show the rest of the distribution. Grey dots show the Δ RT of individual subjects in the experimental group and at the specified time points. * representing *p* ≤ 0.05, ** *p* ≤ 0.01 and *** representing *p* ≤. 001 (Bonferroni corrected for multiple comparisons).

In the next step, we regarded the performance in the learned and novel movement transitions in the new/compatible/incompatible/same sequence in session 4. While the sequence learned in the Same group only consisted of learned transitions and the sequence performed by the New group only consisted of novel transitions, the sequence of the Compatible and Incompatible groups was formed by 4 novel and 4 learned transitions. Moreover, the learned and novel transitions were equivalent between the Compatible and Incompatible groups. Similar to the Δ compound measures, we obtained Δ RTs for novel and learned transitions by contrasting the novel and learned transitions in the sequence condition with the RTs in the random condition, respectively (only correctly executed movement transitions were regarded, thus Δ RTs is equivalent to the Δ compound measures in this case). Figure 3 shows the Δ RTs for learned and novel movement transitions over the course of session 4, separated by experimental group. We split the data into the start and end of session 4 (first and last 4 blocks, respectively) to compare the performance between groups for those time points of interest (see Fig. 4).

For the learned transitions, there was a significant difference in Δ RTs between experimental groups at the start of session 4 (ANOVA: *F* = 8.679, *p* < 0.001). The performance in the Same group significantly differed from the Compatible (*p* < 0.001) and Incompatible (*p* = 0.007) groups, i.e. the performance of the Same group was higher. The Compatible and Incompatible groups showed no difference (*p* = 1.). In contrast, by the end of the session, there was no significant difference in the Δ RTs for learned transitions between groups (ANOVA: 1.721, *p* = 0.189). For the novel transitions, the performance significantly differed between groups at the start of session 4 (Welch’s ANOVA: *F* = 5.769, *p* = 0.006), i.e. the Δ RTs of the New group were significantly lower in comparison to the Compatible (*p* = 0.008) and Incompatible (*p* = 0.034) groups. Furthermore, there was no difference between the Compatible and Incompatible groups (*p* = 1.). At the end of session 4, the difference in the Δ RTs between groups remained significant (Welch’s ANOVA: *F* = 11.987, *p* < 0.001). As for the start of the session, the performance in the New group significantly differed from the Compatible (*p* = 0.002) and Incompatible (*p* = 0.014) groups, with participants in the New group performing worse than in the other groups. The performance between the Compatible and Incompatible groups showed no difference (*p* = 1.).

### Experiment 2 (2 days) - Sequence-specific learning across training sessions

To assess whether the effects in experiment 1 are dependent on the amount of previous training, we performed the same experiment in a 2-day design, where participants train the original motor sequence for only one training session (in contrast to 3 training sessions in experiment 1) before participants had to perform the new, compatible, or incompatible sequence. For the data of the Same group, data from experiment 1 (19 subjects of the Same group) has been used.

**Fig. 5:**
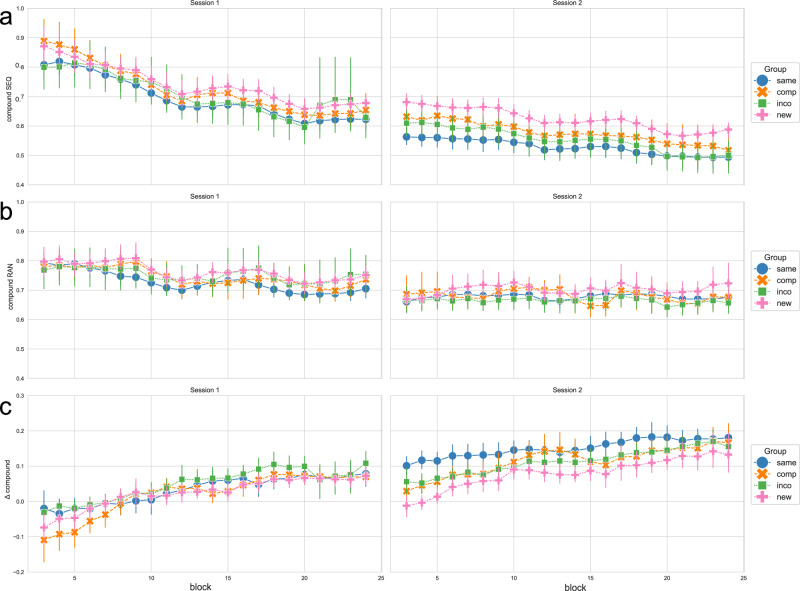
Performance measures in experiment 2 across the 2 training sessions and experimental groups, in the sequence condition, the random condition, and the contrast between both. **a** shows the compound measure, which is based on the RT of correct key presses scaled by the accuracy/number of errors, thus combining RT and accuracy, in the sequence condition (SEQ); **b** shows the compound measure as a combination of RT and accuracy in the random condition (RAN); **c** shows the Δ compound measure, which is obtained by contrasting the random and sequence condition, to obtain a measurement of the performance that is specific to sequence-learning. The experimental groups “same", “comp" (= compatible), “inco" (= incompatible), and “new" are color-coded in blue, orange, green, and pink, respectively. Each column shows a training session. Data is depicted using a sliding window approach (moving average smoothing, *n* = 3). Vertical bars represent the 95 % confidence intervals.

As in experiment 1, the main measurement of learning and performance were the RTs and the accuracy, which we combined into a compound measure (i.e. the RTs of correctly executed sequences were scaled by the accuracy). Figure 5a shows the performance in the implicit sequence learning condition across the 2 training sessions in experiment 2, while Fig 5b shows the performance in the random control condition.

**Fig. 6:**
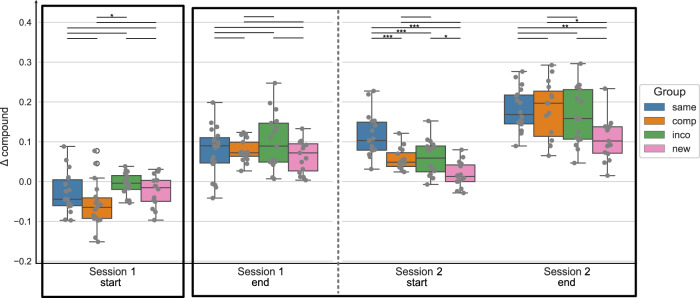
Performance changes specific to sequence learning in experiment 2, represented by Δ compound, at the start and end of each training session. The Δ compound was obtained by contrasting the random and implicit conditions for a sequence-specific learning measure. The first/last 4 blocks of a session are included for the start/end of the session, respectively. The experimental groups “same", “comp" (= compatible), “inco" (= incompatible), and “new" are color-coded in blue, orange, green, and pink, respectively. The data is depicted in box plots or box-and-whisker plots. The box shows the lower and upper quartile of the data, representing 50 % of the compound scores, while the whiskers extend to show the rest of the distribution. Grey dots show the Δ compound of individual subjects in the experimental group and at the specific time points. The black boxes mark the specified timepoints of interest: start of training the initial motor sequence - session 1 start (baseline performance); end of training the initial motor sequence - session 1 end; and session 2 where a new/compatible/incompatible or the same sequence has been trained. At those timepoints, statistical comparisons of the Δ compound measures between the experimental groups were performed (black vertical lines), using paired t-tests, with * representing *p* ≤ 0.05, ** *p* ≤ 0.01 and *** representing *p* ≤ 0.001 (Bonferroni corrected for multiple comparisons).

Figure 5c depicts the Δ compound measures as a proxy for sequence-specific learning over the course of training, for each experimental group. For statistical comparison, we regarded the first and the last 4 blocks of each session, i.e. session 1 start (baseline performance), session 1 end (end of training of the original sequence), session 2 start (early learning of the new/compatible/incompatible/same sequence), and session 2 end (end of training of the new/compatible/incompatible/same sequence), respectively (see Fig. 6).

A mixed ANOVA with the factor timepoint (within-subject) and group (between-subject) revealed a significant effect of the factor timepoint (*F* = 174.331, *p* < 0.001), a significant effect of the factor group (*F* = 4.918, *p* = 0.004) and a significant interaction of timepoint and group (*F* = 4.484, *p* < 0.001) on the Δ compound values (see Supplementary Table 9 for the ANOVA summary). For each experimental group, we performed repeated measures ANOVAs to see if the Δ compound values varied over the course of training. For all groups, there was a significant main effect of the factor timepoint on the performance measure (all *p* < 0.001). The performance significantly improved within session 1, i.e. from session 1 start to session 1 end, in all groups (all *p* < 0.001). However, from session 1 end to session 2 start, the performance significantly decreased in the New group (*p* = 0.003) and in the Compatible group (*p* = 0.005), while it did not significantly differ for the Same group (*p* = 0.245) and the Incompatible group (*p* = 0. 901). The results of the ANOVAs and post hoc comparisons for all groups are shown in Supplementary Tables 11-17. Since we were interested in performance differences across the experimental groups, we performed one-way ANOVAs with the dependent variable Δ compound values and the between-subject factor group, at specific timepoints of interest (the start of session 1 as the baseline performance; the end of session 1 as the end of training the initial motor sequence; and the start and end of session 2, where the New/Compatible/Incompatible or the Same sequence is trained). At the start of the experiment (i.e. session 1 start), there was a main effect of the factor group on the Δ compound values (*F* = 3.614, *p* = 0.018). Post hoc tests revealed that only the performance in the Compatible and Incompatible group significantly differed (*p* = 0.012), with the performance of the Incompatible group being lower than in the Compatible group, while all other comparisons were not significant (see Supplementary Tables 18 and 19). At the end of session 1, there was no significant difference in the Δ compound values across experimental groups (Welch’s ANOVA: *F* = 1.073, *p* = 0.374). Thus, although the baseline performance differed between the Compatible and Incompatible group initially, by the end of the first training session all groups reached a comparable performance.

At the start of session 2, the Δ compound values varied across groups (Welch’s ANOVA: *F* = 14.178, *p* < 0.001).

Post-hoc tests showed a significant difference in Δ compound values between the Same and all other experimental groups (all *p* ≤ 0.001). Moreover, the performance in the Incompatible and New groups differed (*p* = 0.033), i.e. the Incompatible group showed a higher performance, reflected by higher Δ compound values. The performance between the Compatible and New groups (*p* = 0.056) and between the Compatible and Incompatible groups (*p* = 1.) did not differ. Thus, changing the motor sequence from the previously learned one resulted in a deteriorated performance in all groups, while the performance deteriorated more in the Compatible and New groups compared to the Incompatible group. By the end of the training session, the Δ compound values in all experimental groups increased, while there was a significant difference in the performance between groups (*F* = 4.735, *p* = 0.005). Post-hoc tests confirmed a significant difference of the Δ compound values between the Same and New groups (*p* = 0.008) and between the Compatible and New groups (*p* = 0.014), with a lower performance in the New group in both cases. The Same and Compatible groups did not differ (*p* = 1.). The Incompatible group showed no significant difference to the New (*p* = 0.070), the Same (*p* = 1.), and the Compatible groups (*p* = 1.). The results of all statistical analyses on group differences are reported in Supplementary Tables 18-24.

### Experiment 2 (2 days) - Performance in novel versus learned movement transitions

**Fig. 7:**
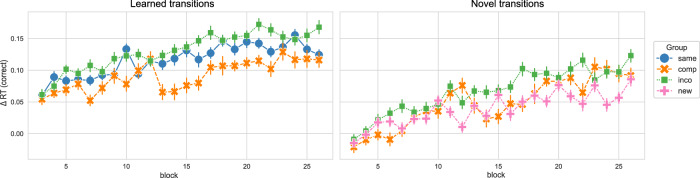
Performance in learned versus novel transitions in experiment 2, over the course of training session 2. The Δ reaction times (RTs) for correctly executed learned and novel movement transitions in experiment 2, across blocks in session 2, separated by experimental groups, are depicted. The Δ RT was obtained by contrasting the random and implicit conditions for a sequence-specific learning measure. While both the Compatible and Incompatible sequences contain 50 % learned and 50 % novel transitions, the Same sequence contains only learned transitions, and the New sequence contains only novel transitions. The experimental groups “same", “comp" (= compatible), “inco" (= incompatible), and “new" are color-coded in blue, orange, green, and pink, respectively. Vertical bars represent the 95 % confidence intervals.

**Fig. 8:**
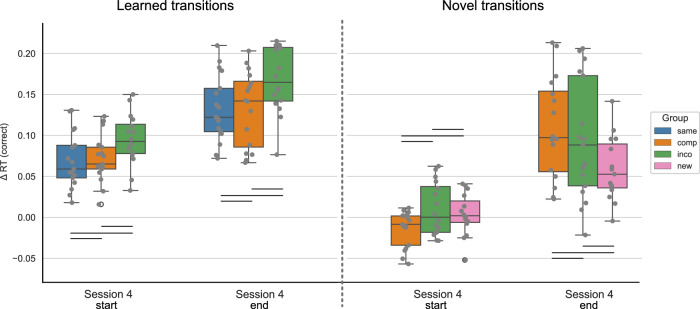
Performance in learned versus novel transitions in experiment 2, at the start and end of session 2. The Δ reaction times (RTs) for correctly executed learned and novel movement transitions in experiment 2, at the start and end of session 2, separated by experimental groups, are depicted. The Δ RT was obtained by contrasting the random and implicit conditions for a sequence-specific learning measure. The first/last 4 blocks of a session are included for the start/end of the session, respectively. While both the Compatible and Incompatible sequences contain 50 % learned and 50 % novel transitions, the Same sequence contains only learned transitions, and the New sequence contains only novel transitions. The experimental groups “same", “comp" (= compatible), “inco" (= incompatible), and “new" are color-coded in blue, orange, green, and pink, respectively. The data is depicted in box plots or box-and-whisker plots. The box shows the lower and upper quartile of the data, representing 50 % of the RT scores, while the whiskers extend to show the rest of the distribution. Grey dots show the Δ RT of individual subjects in the experimental group and at the specified time points, with * representing *p* ≤ 0.05, ** *p* ≤ 0.01 and *** representing *p* ≤ 0.001 (Bonferroni corrected for multiple comparisons).

To estimate retention and integration of old and new information, we split the RT data of session 2 into learned and novel transitions (shared with or non-existent in the original motor sequence, respectively). The RTs contrasted with the RTs of the random sequences, i.e. the Δ RTs, specifically for the learned and novel transitions are shown in Fig. 7. For statistical comparison, we regarded the Δ RTs of novel and learned transitions at the start and the end of session 2 (see Fig. 8). For learned transitions, a one-way ANOVA revealed a significant effect of the factor group on the Δ RTs (*F* = 3.291, *p* = 0.046) at the start of session 2, while post hoc analyses showed no significant difference in the performance between all groups (Compatible and Incompatible: *p* = 0.118, Compatible and Same: *p* = 1., Incompatible and Same: *p* = 0.073). Similarly, at the end of training session 2, a significant main effect of the factor group was found on the Δ RTs of novel transitions (*F* = 3.716, *p* = 0.032). However, none of the post hoc comparisons was significant (Compatible and Incompatible: *p* = 0.070, Compatible and Same: *p* = 1., Incompatible and Same: *p* = 0.063).

For novel transitions, the performance at the start of session 2 significantly differed across groups (*F* = 3.513, *p* = 0.038), i.e. the Δ RTs of the Compatible and Incompatible group showed a significant difference (*p* = 0.043), with reduced performance in the Compatible group. Neither the performance in the Compatible and New (*p* = 0.212) nor in the Incompatible and New groups (*p* = 1.) significantly differed. However, the Δ RTs of the Compatible group showed a non-normal distribution at the start of session 2, as probed with a Shapiro-Wilk test (*p* = 0.028, please see Supplementary Table 25). Thus, we performed a Kruskal-Wallis test as a non-parametric alternative on the Δ RTs for novel transitions at the start of session 2. The test revealed no significant difference between experimental groups (Kruskal-Wallis test statistic: 4.067, *p* = 0.131). Despite a significant main effect of the factor group on the Δ RTs of novel transitions at the end of session 2 (Welch’s ANOVA: *F* = 2.796, *p* = 0.027), none of the post hoc comparisons showed a significant difference (Compatible and Incompatible: *p* = 1., Compatible and New: *p* = 0.077, Incompatible and New: *p* = 0.320). Overall, the performance for novel transitions in the Compatible, Incompatible, and New groups and the performance for learned transitions in the Compatible, Incompatible, and Same groups showed no significant differences at the start or end of session 2.

## Discussion

In this study, we investigated in an implicit motor sequence learning (MSL) task, if learning new sequences can be facilitated if the new sequence is compatible with a previously learned one. Moreover, conducting two experiments that varied only in the number of training sessions, we investigated whether the amount of previous training influenced the effect of facilitative learning.

In summary, all experimental groups showed performance improvements in the bimanual SRTT over the course of practicing the original motor sequence. Switching from the original motor sequence to a new sequence (New group), lead to a deterioration in performance, independent whether the original sequence has been practiced for 3 days (experiment 1) or for 1 day (experiment 2). Similarly, performing the compatible and incompatible sequence lead to a decrease in performance in the Compatible and Incompatible groups compared to the Same group. However, at least in experiment 1 (3 days of prior training), the performance in the Compatible and Incompatible groups was significantly better than the performance in the New group. In experiment 2, this was only the case for the Incompatible group. By the end of the last training session (session 4 in experiment 1, session 2 in experiment 2), the New group, practicing the new sequence over the course of one training session, could not reach the performance of the Same group, that continued to practice the original sequence. However, the Compatible and Incompatible groups showed a similar performance to the Same group by the end of the training session, in experiment 1 and in experiment 2. Moreover, the performance of the Compatible and Incompatible groups were comparable (i.e. no significant difference) across all timepoints of learning, with the exception of the baseline performance at the start of session 1 in experiment 2. Notably, even given the different starting point of learning between the Compatible and Incompatible groups, both reached a similar performance before and after the intervention. Regarding the performance in learned and novel movement transitions (shared and not shared with the original sequence, respectively), allows us to investigate the retention of old and the integration of new information. In experiment 2, there was no significant difference between the performance for learned transitions in the Same, Compatible, and Incompatible groups. Similarly, there was no significant difference in the performance for novel transitions between the Compatible, Incompatible, and New groups. In contrast, in experiment 1, where the original sequence was practiced for 3 sessions before the intervention, the performance for learned transitions was initially reduced in the Compatible and Incompatible groups compared to the Same group. By the end of session 4 however, the performance in the learned transitions showed no significant difference between the Compatible, Incompatible, and Same groups. Interestingly, the performance for novel transitions in the Compatible and Incompatible groups was higher compared to the New group.

The underlying basis for our study was work by King et al.^17^ on schema memory in explicit MSL, i.e. sequences compatible in ordinal positions with a previously learned sequence, were learned faster via rapid integration of the new information. The sophisticated study design used in King et al.^17^ allowed testing whether compatibility in ordinal positions or movement transitions influenced the learning of new motor sequences, since the sequences in the Compatible/Incompatible group only differed in terms of their overlap in ordinal positions with the previously learned sequence (high/low ordinal overlap, respectively), while the movement transitions were the same. Importantly, our study design differed as follows: Firstly, we used an implicit version of the bi-manual SRTT, with no instructions about the presence of a sequence, thus, learning was incidental. Secondly, in experiment 1, participants trained the original motor sequence for 3 consecutive days before switching to the new/compatible/incompatible sequence on the following day in session 4. Moreover, we included random sequence trials in each training block, which allowed us to extract sequence-specific performance gains, independent of general task-practice effects, motivation, and fatigue^15,16^. While in the explicit task by King et al.^17^ RTs and accuracy were analyzed separately, we combined the primary measurement of learning, the reaction times (RTs), and the accuracy, into a compound measure also referred to as inverse efficiency^27^. Accuracy is assumed to relate more to the explicit component^12^ of MSL and played a secondary role in our design.

Overall, our study (i.e. experiment 1) indicates that the learning of new sequences that were compatible with a previously learned one in terms of movement transitions (i.e. the compatible and incompatible sequences) was facilitated, since both the compatible and incompatible sequences were executed with a higher performance than a completely new sequence and both the Incompatible and Compatible group reached the performance level of the Same group by the end of the experiment. At the same time, there was no difference in the performance between the Compatible and Incompatible group evident, thus the determining factor of compatibility is not the ordinal position, but rather the movement transitions shared with the previously learned sequence. In contrast, King et al.^17^ reported facilitative learning of new sequences that were compatible in terms of ordinal positions with a previously trained sequence in their explicit MSL task.

Learning in MSL and the SRTT involves two learning systems, as stated in the Dual-System model^28^. Firstly, a multidimensional system that associates representations from multiple dimensions (motor and higher-order representations such as sequence order), supporting explicit MSL (see also: Willingham^28^)^29^. The second system is unidimensional or intra-dimensional and associates representations within one dimension^28^. This could represent implicit MSL via the association of subsequent motor responses. Similarly, Verwey et al.^30^ proposed a framework consisting of a motor processor that relies on motor representations and a central processor that uses central-symbolic representations. The latter could relate to abstract representations such as ordinal positions of sequence elements. Our results, in combination with the findings from King et al.^17^, align with those models on sequence representations: For explicit MSL, a cognitive-motor schema is built based on the ordinal positions (King et al.^17^), while in implicit MSL movement transitions form the basis for an associative map.

It is of particular interest that the performance in the learned transitions initially deteriorated in the compatible and incompatible groups. This might relate to memory reactivation processes, where the retrieved memory trace is temporarily rendered labile and sensitive to interference or reinforcement before re-consolidation^31,32^. For the compatible and incompatible sequences, the previously learned motor memory might be updated by associating the learned movement transitions with the novel ones. Re-consolidation has been previously reported in motor skill learning^33^, i.e. the performance of a motor sequence in a finger-tapping task was impaired after learning a second, interfering sequence^33^, although this effect could not be sufficiently replicated^34^.

Since implicit MSL is also referred to as the slow component of MSL and relies on practicing the task^11,12^, we included a second experiment to test whether the facilitative learning effect from experiment 1 depends on the amount of previous training. In experiment 2, participants trained the original sequence only for one session before performing the altered sequence in session 2. Although the data of experiment 1 and 2 share some common patterns (deteriorated performance in all groups compared to the Same group when the compatible/incompatible/new sequence is introduced), there are some notable distinctions in the results of both experiments. While in experiment 1 the performance of the Compatible and Incompatible groups was reduced, there was still a significant difference to the even more deteriorated performance of the New group (at the start and at the end of the last training session). In experiment 1, at the start of the last training session, the performance of the Incompatible group was higher compared to the New group, while there was no difference in the performance of the Compatible and New group. At the end of the last training session, the Compatible group showed higher performance scores than the New group, while the performance did not differ between the Incompatible and New groups. On the contrary, the Same group had a higher performance compared to the New group at both timepoints. Moreover, although the performance for learned transitions was comparable across the Same, Compatible and Incompatible group, the Compatible and Incompatible groups failed to show an enhanced performance in novel transitions in comparison to the New group. Thus, the data points to the assumption that retention of learned transitions enhanced the performance in the Compatible and Incompatible groups compared to the New group, but there was no enhanced integration process of novel transitions, in experiment 2.

The effect of previous practice on skill memory consolidation, i.e. delayed between-session performance gains in the absence of training, has been reported previously^35^. Only when participants trained long enough for the within-session performance gains to saturate, consolidation processes were triggered^35^. In experiment 1, the performance gains in session 3 were minimal, thus, our results align with this assumption and explain the difference to the results from experiment 2 (only 1 session of previous training). In our study, 1 session resulted in 288 repetitions of the sequence (in the ideal scenario of an accuracy of 100%). Other SRTT studies used for example 80 sequence repetitions (Nissen and Bullemer^14^). In another implicit MSL task, 192 sequence repetitions were used for practice, before an intervention (alteration of order or timing) was performed^22^. Thus, the number of practice trials in our design is not unusually low, still, one training session might have been too low to induce any consolidation processes. We have chosen the number of trials and sessions in experiment 2 in consistence with the study by King et al.^17^. However, given that implicit learning is characterized by slow and gradual performance changes, we wanted to have a higher number of training session in experiment 1 by including multiple training sessions of the original sequence. We chose the amount of practice in a way, that participants reached an intermediate learning stage and an asymptotic or almost asymptotic learning curve before switching to the new/compatible/incompatible sequence. Another study investigating the effector transfer in motor sequences has for example used 1 day of training with 160 repetitions versus 4 days of training (640 repetitions in total)^36^. In the Discrete Sequence Production (DSP) task, another paradigm to study motor sequence learning, typically 500-1000 sequence trials are used to study intermediate learning and the building of motor chunks^37,38^. Thus, in our study design, we also aimed for at least 500-600 sequence repetitions before switching to the New/Compatible/Incompatible sequence in experiment 1.

Another important aspect of any implicit MSL task is the effect of explicit learning or declarative knowledge. The SRTT being a purely implicit learning task is highly unlikely and has been challenged before^16^. Moreover, we use the SRTT as a motor learning task, although also perceptual learning is known to play a role^15,39^.

In MSL tasks, explicit or declarative knowledge about the sequence regularities is typically probed at the end of learning, for example in sequence generation or recall tests^14,40^. However, testing explicit knowledge or the awareness of sequence regularities remains challenging since the tests lack specificity and sensitivity^15,41^. Moreover, it is not feasible to infer declarative knowledge during learning from tests at the end of training since awareness is subject to decay over time^42^. Notably, once the declarative knowledge of participants is probed via recall tests, their attention might shift towards regularities in the presented stimuli in subsequent blocks or sessions. This poses a tremendous issue in multi-session implicit learning tasks. Especially the prolonged practice of the Same sequence in experiment 1 (4 training sessions for the Same group, 3 training sessions for all other groups) makes it more likely that some participants developed explicit awareness of the underlying sequence (partially or fully). We aimed to keep participants naive about the repeating sequence throughout all sessions, thus it was not possible to test the declarative knowledge during training. Instead, our analysis relied on ongoing performance to measure implicit learning. Although we can not exclude that some of the individual subjects gained awareness about the repeating sequence over the course of training, on a group level, the performance measures showed slow and gradual changes (decrease for RTs, increase for compound scores) which is characteristic for implicit learning in the SRTT (see also Dyck and Klaes^26^). Moreover, although we adapted the task design of King et al.^17^ (implicit version of their explicit task), our data differed from their reported data: While in the study of King et al.^17^ the Compatible group performed significantly better than the Incompatible group, we found no significant difference in the compound measures between the Compatible and Incompatible groups (except for the baseline performance at the start of session 1, in experiment 2). Considering that implicit learning is based on associations of motor representations between consecutive button presses^28^ and the incompatible and compatible sequence share the same movement transitions, this further emphasizes implicit learning in our study.

To conclude, our study reported facilitative learning of new motor sequences in implicit MSL, if the sequences were compatible in terms of movement transitions, while this effect relied on a sufficient amount of previous training. Hereby, novel movement transitions were integrated faster in the previously learned representations. The reported effect is similar to the schema memory effect reported in declarative memory and explicit MSL. Nonetheless, since the associative map built is likely based on simple associations between consecutive movements within a single dimension, we prefer the expression facilitative learning or schema-like memory effect.

Our study adds to the current literature on transfer learning of implicit MSL, showing that under specific conditions (sufficient amount of previous training, compatibility between trained and new content) the learning of new, compatible motor sequences can be facilitated. Partial transfer of implicit MSL to new learning context (altered stimulus order or timing) has been reported previously^22^. Extracting the crucial factors that determine if learning under new (similar) conditions will be successful, can be advantageous not only in optimizing for example training schedules in the context of sport but also in rehabilitation scenarios.

## Materials and Methods

### Participants

In total, 142 subjects participated in the study: 87 subjects in Experiment 1, and 55 subjects in Experiment 2. In experiment 1, the data of 16 subjects had to be excluded due to not completing all training sessions or due to technical issues that occurred during data storage. The data of the remaining 71 subjects in Experiment 1 is reported. Table 1 shows the distribution of participants within the experimental group, including their mean age and sex. Inclusion criteria for this study were: age between 18 and 35 years, normal or corrected-to-normal vision, no psychological or neurological disorders, and no sleep disorders. None of the participants stated being an advanced or professional musician, engaging in sports at a professional level (i.e. more than 5 hours per week), or playing computer or console games for more than 5 hours per week. Before participation, all subjects gave written informed consent in accordance with the Declaration of Helsinki. The study received ethical approval (associated with the approval number 405, Research Ethics Board of Psychology, Ruhr-University Bochum). All subjects were compensated for their participation through course credits.

**Table 1:** Summary of the participant characteristics in Experiment 1 and 2: number of subjects per group (n), mean age in years and the standard deviation, and number of female and male subjects

Experiment 1			
Experimental group	n	mean age [years]	female/male
Same	19	24.7 ± 4.8	16/3
Compatible (comp)	18	22.2 ± 2.7	15/3
Incompatible (inco)	17	23.8 ± 3.7	13/4
New	17	23.1 ± 2.9	16/1

Experiment 2			
Experimental group	n	mean age [years]	female/male
Compatible (comp)	19	24.7 ± 5.0	14/5
Incompatible (inco)	18	25.7 ± 5.3	12/6
New	18	25.7 ± 4.3	14/4

### Motor sequence learning task

The participants performed a bimanual version of the serial reaction time task (SRTT)^14^ where visual stimuli cue key presses in a forced choice reaction time task. The motor sequence learning (MSL) task was designed similar to the study of King et al.^17^ (specifically experiment 1a in their study) with the major distinction that participants were not informed about the presence of a repeating sequence, thus realizing an implicit version of the explicit motor task of King et al.^17^. Eight squares were presented on the screen, aligned horizontally, while their spatial arrangement matched the position of the eight fingers of both hands (excluding thumbs) used for the motor response. Initially, at the start of each block, the squares had a grey fill color and a red outline, indicating a break or no movement phase to the participant. After 5 seconds, the outline changed from red to green signaling the participant to prepare for the upcoming stimulus. After another 2 seconds, one of the squares changed its (fill) color from grey to green, indicating that the corresponding key should be pressed, using the corresponding finger. Following a motor response, the next stimulus immediately lit up in green, triggering the next motor response. Importantly, the trial continued with any motor response, even if an incorrect key was pressed and no feedback was provided if the key press was correct or not. There were two experimental conditions, determining the order of the presented stimuli: Either the stimuli were presented in a pseudo-random order, representing the random condition, or the stimuli followed a fixed repeating sequence, representing the implicit sequence condition. Each sequence trial consisted of eight key presses, with each of the eight keys occurring once, in the implicit sequence condition and for random sequences. A “sequence" block consisted of 12 repetitions of the implicit fixed 8-element sequence, interspersed with four random sequences (for a total of 16 sequence trials) in the following order: 1x random, 4x sequence, 1x random, 4x sequence, 1x random, 4x sequence, 1x random. On the one hand, this design allows us to track the performance of random sequences beside the implicit sequence as an indicator for general performance improvements that are not specific to sequence learning, or factors such as motivation and fatigue^15,16^. On the other hand, we aimed to minimize the likelihood of participants noticing the repeatedly occurring sequence by framing the sequences into random sequence trials, especially at the start and the end of each block. There was no break period between sequence trials and in total, one block resulted in 128 (16 times 8) key presses.

### Experimental Design

The experiment was programmed in PsychoPy^43^, converted to JavaScript, and conducted as an online study via PsychoPy’s Pavlovia (see Bridges et al.^44^). Participants were informed that the study is on hand-eye coordination and that they will train a reaction time experiment in multiple sessions on consecutive days, i.e. 4 days for experiment 1 and 2 days for experiment 2. Before the start of the experiment, participants filled out a consent form and a short questionnaire that collected information on their age and gender, whether they played any musical instruments or computer/console games, and the number of hours they engaged in sports per week. Subsequently, they started the first experimental session at a time and date of their choice with the restriction to perform all sessions at approximately the same time of day to reduce confounding effects of the circadian phase variation on behavior^45^. The subjects were instructed to respond to the visual cues by pressing the spatially congruent key as fast and as accurately as possible (i.e. with an emphasis on pressing the correct key). At the start of each session, a short demo was performed, where each finger had to be pressed once from left to right to ensure correct placement of the fingers on the keyboard. To familiarize the participant with the experiment, each session started with 2 blocks of only random sequences, i.e. 16 random sequences per block. Afterward, 24 “sequence" blocks followed (12 repetitions of the implicit sequence and 4 random sequences per block). After blocks 9 and 17, the opportunity for a break was provided, while the participant decided individually when to resume the experiment. In experiment 1, participants underwent 4 experimental sessions on 4 consecutive days. The first 3 sessions were the same for all subjects; i.e. independent of the experimental group, all subjects trained the same repeating sequence. While the experimental procedure in session 4 remained the same as in sessions 1-3, the implicit sequence was changed in this final training session depending on the experimental group: The New group learned a new motor sequence, in which only 2 of the 8 elements remained at the same ordinal position as in the previously learned sequence (low ordinal compatibility). As a result, all the movement transitions between consecutive button presses in the new sequence were novel. Subjects in the Compatible group trained a sequence where 2 elements from the originally trained sequence were exchanged, such that 6/8 elements remained at their previous ordinal position (high ordinal compatibility). In this compatible sequence, 4 movement transitions remained the same (learned transitions) and 4 movement transitions were new (novel transitions). Subjects in the Incompatible group learned a shifted version of the compatible sequence, resulting in the same number of learned/novel movement transitions as the compatible sequence and a very low ordinal compatibility with the initially learned sequence. Importantly, the terminology Compatible and Incompatible groups/sequences refer only to compatibility in ordinal positions. We used the same terminology as in the study by King et al.^17^ for consistency reasons. Regarding movement transitions, the compatible and incompatible sequences are equally compatible - they do not only share the same number of novel/learned transitions but the same learned and novel movement transitions. The novel movement transitions from the compatible and incompatible sequences were identical to 4 of the 8 novel movement transitions in the new sequence. Finally, the Same group continued to train the same sequence of key presses from the previous sessions, which served as a control condition (maximal ordinal compatibility and number of learned movement transitions). By comparing the performance between experimental groups when the manipulated sequence is introduced, we can investigate if there is any retention effect (in the learned transitions in the Compatible and Incompatible groups compared to the Same group), and if such an effect depends on the compatibility in terms of ordinal position or in terms of movement transitions (by comparing the performance of the Compatible and Incompatible group). Furthermore, regarding the novel movement transitions in the Compatible and Incompatible groups in comparison to the New group, allows us to investigate the integration processes of novel transitions (as also described by King et al.^17^). Thus, the New and Same groups served as control groups representing the two extreme cases, i.e. a completely new versus the same sequence as the original one. The two experimental groups Compatible and Incompatible are the groups of interest. The performance in those groups is compared to the New and Same groups to infer if there is any facilitative learning effect in one or both of the groups. A facilitative effect would be reflected by an improved performance in comparison to the New group and a similar performance than the Same group. Moreover, the comparison between the two groups of interest, i.e. the Compatible and Incompatible group, allows us to infer whether the ordinal structure or the movement transitions play a role in facilitative learning of similar motor sequences.

**Fig. 9:**
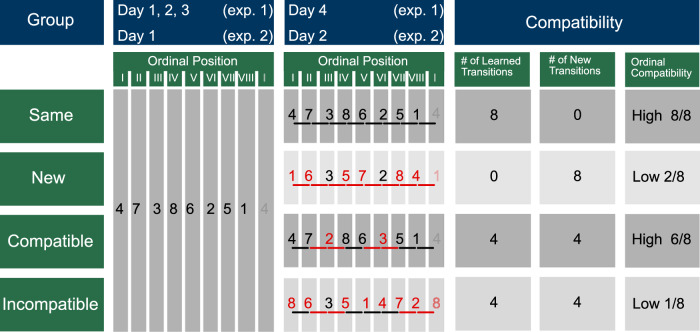
Experimental design, showing the experimental groups and respective motor sequences for each training session. Each row represents the following data for one experimental group: group label (1st column); the sequence of key presses learned during days 1-3 in experiment 1 and during day 1 for experiment 2 (2nd column); the motor sequence learned in day 4 for experiment 1 and day 2 for experiment 2 (3rd column) - here, elements with the same ordinal position as in the original sequence are depicted in black, while elements not corresponding to the original sequence in terms of ordinal position are depicted in red. Moreover, transitions between elements that are preserved from the original sequence are depicted in black, while new movement transitions are depicted in red; columns 4-6 show the compatibility between the motor sequence of the initial training session(s) and the last training session: column 4 ('# of Learned Transitions') shows the number of learned or old transition, which reflects the compatibility in terms of movement transitions; accordingly, column 5 ('# of New Transitions') shows the number of new movement transitions; lastly, column 6 ('ordinal compatibility') shows the compatibility in terms of ordinal positions. This figure showing the experimental design was replicated and adapted from King et al.^17^.

Experiment 2 followed the same experimental setup with the distinction of only 2 experimental sessions on 2 consecutive days. Here, participants trained an implicit sequence for one session, while in session 2 they trained another implicit sequence based on their experimental group (New, Compatible, Incompatible). The movement sequences used in experiments 1 and 2 were identical. In experiment 2, there was no Same group, since it was redundant given the data of experiment 1, where participants trained the same sequence for 4 days. Thus, the data of 19 subjects from the Same group (first 2 sessions) in experiment 1 has been used as the Same group in experiment 2. Participation in experiment 1 excluded subjects from participating in experiment 2 and vice versa, such that each participant performed the motor sequences only the number of times that was specified by the study design. An overview of the experimental design, displaying the movement sequences for each session and experimental group, is shown in Fig. 9. Comparing the results of experiments 1 and 2 allows us to investigate if the amount of previous practice plays a role in learning a new motor sequence.

### Data processing and statistical analysis

The primary measurements recorded were the reaction time (RT) and the accuracy as the number of correct key presses divided by the number of sequence elements. The mean RT per sequence trial and the accuracy were computed per block for each experimental session. Furthermore, a compound measure combining the RT and the accuracy was calculated based on the following formula:

1 $$compound=\frac{R{T}_{correct}* 100}{accuracy}$$

Thus, the compound measure for one block is the RT of correctly executed sequences within that block scaled by the accuracy (% correct) in that block, also referred to as inverse efficiency^27^. Since every block consisted of 12 repetitions of the implicit sequence and 4 repetitions of random sequences, this metric was computed for the sequence and random conditions, respectively. Performance improvements in the random condition capture general practice effects such as a strengthened association between the visual cues and the motor responses, which is not specific to sequence learning. To extract performance gains that are specific to sequence learning, we contrasted the compound measures between the implicit and the random condition (similar to the RT contrasting approach in Pollok et al.^25^ and Dyck and Klaes^26^):

2 $$\Delta compound=compoun{d}_{{\rm{random}}}-compoun{d}_{{\rm{implicit}}}$$

The subtraction is done block-wise, individually for each subject. Outlier removal was performed based on the z-score threshold (z=2). For statistical comparisons, the Δ compound values within the first 4 blocks and the last 4 blocks in each session were averaged as a performance measure at the start and end of the session, respectively. The resulting Δ compound values were used to perform a mixed ANOVA with timepoint as the within-subject factor (e.g. session 1 start, session 1 end, session 2 start, etc.) and the experimental group as the between-subject factor (Same, New, Compatible, Incompatible group). In case sphericity was not met (probed by Mauchly’s test of sphericity), a Greenhouse-Geisser correction was applied. In case of a significant main effect of timepoint, we compared the data of all timepoints for one experimental group, thus performing a one-way repeated measures ANOVA with the within-subject factor timepoint, allowing us to investigate if the performance of the Same, New, Compatible or Incompatible group changed over the course of training (within one training session and across training sessions). Moreover, we were interested in whether the performance differed across experimental groups at the various timepoints, which we probed with one-way ANOVAs with the between-subject factor group. Specifically, the time points of interest in experiment 1 were: the start of session 1, marking the start of the training and thus the baseline performance; the end of session 3, representing the end of training of the initial motor sequence; the start and the end of session 4, representing the initial learning of the new/incompatible/compatible/same sequence and the performance at the end of the experiment, resulting from online learning during one training session, respectively. Accordingly, in experiment 2 the time points of interest were: the start of session 1 for the baseline performance, the end of session 1, and the start and end of session 2. If the equality of variances was violated (Levene’s test), Welch’s ANOVA was used instead. In case of a significant main effect, pairwise t-tests were performed for post hoc analysis, applying Bonferroni correction for multiple comparisons. We chose this approach instead of directly performing post hoc tests on the mixed ANOVA to reduce the number of comparisons (for experiment 1 8 timepoints and 4 experimental groups resulted in a total of 496 comparisons; for experiment 2 4 timepoints and 4 experimental groups resulted in 120 comparisons). In the next step, we analyzed the movement transitions in the new/compatible/incompatible/same sequence for learned/old versus novel transitions. While the motor sequence performed by the Same group only contains previously learned movement transitions, the New group executed only novel movement transitions. In contrast, both the compatible and incompatible sequences shared 4 learned movement transitions with the originally learned sequence (i.e. 4-7, 8-6, 5-1, 1-4), while the other 4 transitions were novel (see Fig. 9). Importantly, all transitions in the compatible and incompatible sequence were shared, since the incompatible sequence is essentially a shifted version (in terms of ordinal positions) of the compatible sequence. Regarding the performance in the learned versus novel transitions, retention and integration of the old/new information can be inferred, following the study of King et al.^17^. We only included correctly executed learned and novel movement transitions in our analyses, thus focusing on the RT. As for the compound measure, Δ RT was calculated by contrasting the RT in the sequence condition with the RT in the random condition to estimate the sequence-specific performance:

3 $$\Delta RT=R{T}_{{\rm{random}}}-R{T}_{{\rm{implicit}}}$$

The subtraction is done block-wise, individually for each subject. Δ RT is calculated separately for learned and novel transitions, while for *RT*_random_ all correctly executed movement transitions within that block are used. To statistically compare the performance for novel and learned transitions across groups, we focused on the Δ RTs at the start (first 4 blocks) and end (last 4 blocks) of the last training session (session 4 in experiment 1, session 2 in experiment 2). Following the same procedure as for the Δ compound values, we performed a one-way ANOVA comparing the Δ RTs across groups (between-subject factor). We performed the ANOVA for each condition (Learned, Novel) and for the two timepoints (start of session 4, end of session 4). In case of a significant main effect, post hoc analysis was performed using pairwise t-tests. The reported p values are adjusted for multiple comparisons using Bonferroni correction. An *α* level of 0.05 was used for all statistical tests.

To test the normality of the data (i.e. the Δ compound measures and the Δ RTs for the Learned/Novel transitions), Shapiro-Wilk tests have been performed. The results can be found in the Supplementary material (Supplementary Table 2 and 10 for the Δ compound measures, Supplementary Table 8 and 25 for the Δ RTs for the Learned/Novel transitions, for experiment 1 and 2, respectively). If the assumption of normality was violated, we also performed non-parametric tests, which are reported in the Supplementary material.

For data processing and statistical analysis, we used Python, including the packages scipy^46^, pingouin^47^, and scikit-posthocs^48^, as well as the software JASP^49^.

## Supplementary information

Supplementary Material

## Data Availability

All data used in the current study is available from the corresponding author upon reasonable request.

## Code availability

The underlying code for analyzing the data may be made available to qualified researchers upon reasonable request from the corresponding author.
