# Supplementary material for: Faster implicit motor sequence learning of new sequences compatible in terms of movement transitions
Source: NPJ Sci Learn. 2025 Jan 16;10:3. doi: 10.1038/s41539-025-00296-4 (PMC11739496; doi:10.1038/s41539-025-00296-4)
Supplement: Supplementary file 1 — Supplementary Material [file 41539_2025_296_MOESM1_ESM.pdf]

# Faster implicit motor sequence learning of new sequences compatible in terms of movement transitions


## SUSANNE DYCK[1,2,*] AND CHRISTIAN KLAES[1,2,3,*]

[1] *Department of Neurotechnology, Medical Faculty, Ruhr-University Bochum, Universitaetsstrasse 150, Bochum, 44801, Germany*
[2] *International Graduate School of Neuroscience, Ruhr-University Bochum, Universitaetsstrasse 150, Bochum, 44801, Germany*
[3] *Neurosurgery, University hospital Knappschaftskrankenhaus Bochum, In der Schornau 23-25, Bochum, 44892, Germany*
[*] *susanne.dyck@rub.de, christian.klaes@rub.de*


## SUPPLEMENTARY MATERIAL

## 1. RESULTS: EXPERIMENT 1 (4 DAYS)

### A. Performance measures: reaction times and accuracy

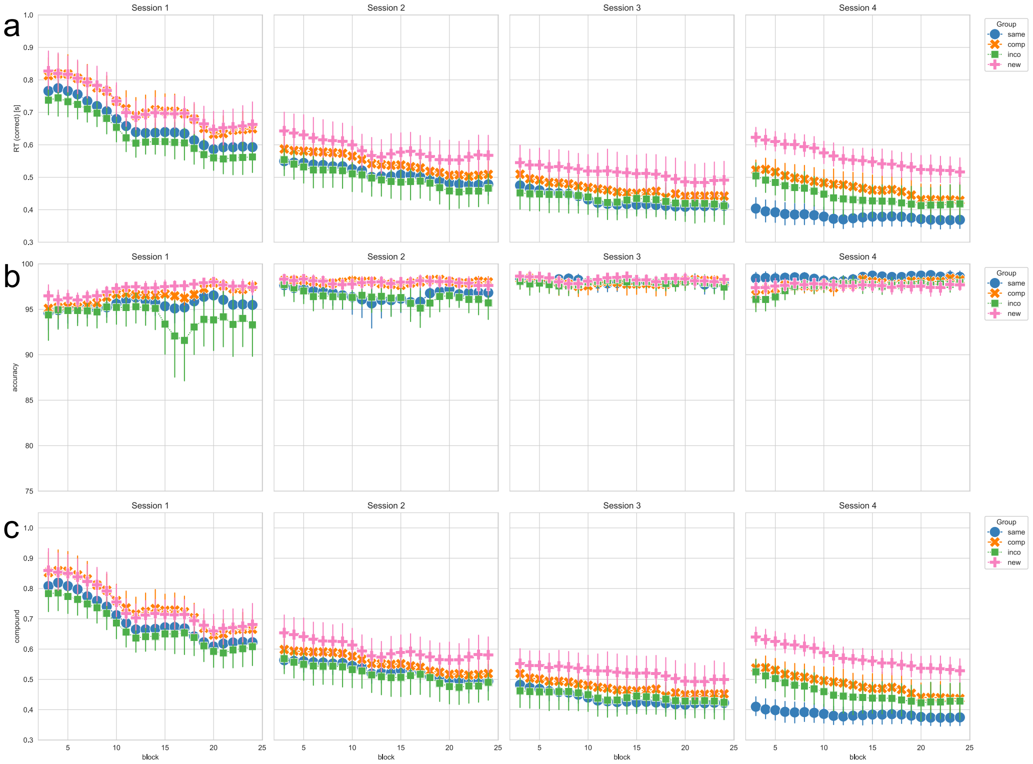


**Supplementary Figure 1.** Performance measures in the SEQUENCE condition in experiment 1 across the 4 training sessions and experimental groups. a) shows the reaction time (RT) for correctly executed sequences; b) shows the accuracy in terms of the number of correct key presses divided by the number of sequence elements (8); c) shows the compound measure which is based on the RT of correct key presses scaled by the accuracy/number of errors, thus combining RT and accuracy. The experimental groups "same", "comp" (=compatible), "inco" (=incompatible), and "new" are color-coded in blue, orange, green, and pink, respectively. While each row shows a specific behavioral measure (RT, accuracy, compound measure), each column shows a training session. Data is depicted using a sliding window approach (moving average smoothing, n=3). Vertical bars represent the 95 % confidence intervals.

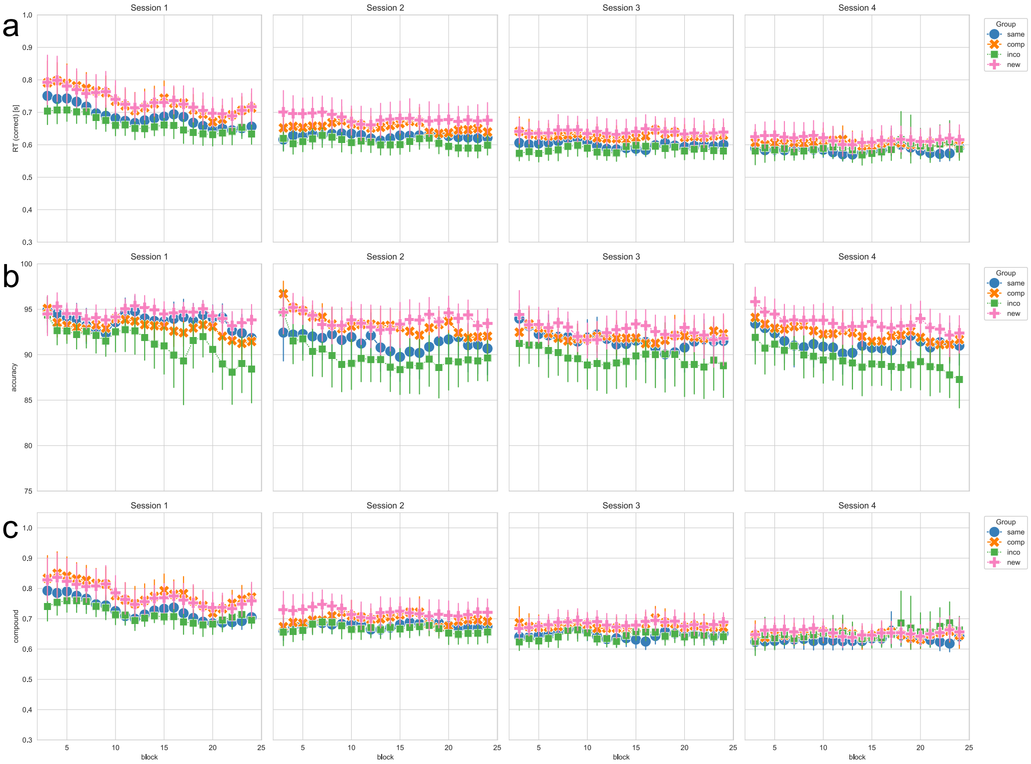


**Supplementary Figure 2.** Performance measures in the RANDOM condition in experiment 1 across the 4 training sessions and experimental groups. a) shows the reaction time (RT) for correctly executed sequences; b) shows the accuracy in terms of the number of correct key presses divided by the number of sequence elements (8); c) shows the compound measure which is based on the RT of correct key presses scaled by the accuracy/number of errors, thus combining RT and accuracy. The experimental groups "same", "comp" (=compatible), "inco" (=incompatible), and "new" are color-coded in blue, orange, green, and pink, respectively. While each row shows a specific behavioral measure (RT, accuracy, compound measure), each column shows a training session. Data is depicted using a sliding window approach (moving average smoothing, n=3). Vertical bars represent the 95 % confidence intervals.

## B. Statistical analysis - Δ compound measure

**Supplementary Table 1. Output of the mixed ANOVA of the Δ compound values with the timepoint as a within-subject factor and the experimental group as a between subject factor.** The timepoints in experiment 1 consisted of: session 1 start, session 1 end, session 2 start, session 2 end, session 3 start, session 3 start, session 4 start, session 4 end. Mauchly's test of sphericity indicated that the assumption of sphericity was violated (p<.001), thus, a Greenhouse-Geisser correction (GG) has been applied. The analysis was performed in JASP [1].

**Within Subjects Effects**

| Cases | Sphericity Correction | Sum of Squares | df | Mean Square | F | p |
|---|---|---|---|---|---|---|
| timepoint | GG | 2.127 | 4.571 | 0.465 | 129.568 | < .001 |
| timepoint * group | GG | 0.259 | 13.712 | 0.019 | 5.256 | < .001 |
| Residuals | None | 0.854 | 364.000 | 0.002 | | |

**Between Subjects Effects**

| Cases | Sum of Squares | df | Mean Square | F | p |
|---|---|---|---|---|---|
| group | 0.241 | 3 | 0.080 | 5.442 | 0.002 |
| Residuals | 0.767 | 52 | 0.015 | | |

**Supplementary Table 2. Output of the normality tests (Shapiro-Wilk test) of the Δ compound values for each group and each timepoint.** The timepoints in experiment 1 consisted of: session 1 start, session 1 end, session 2 start, session 2 end, session 3 start, session 3 start, session 4 start, session 4 end. The analysis was performed in Python using the scipy package [2].

| | timepoint | group | Shapiro-Wilk Test Statistic | p-value |
|---|---|---|---|---|
| 0 | session 1 start | comp | 0.946260 | 0.369391 |
| 1 | session 1 end | comp | 0.943533 | 0.332671 |
| 2 | session 1 start | inco | 0.905602 | 0.098857 |
| 3 | session 1 end | inco | 0.968454 | 0.790820 |
| 4 | session 1 start | new | 0.961360 | 0.657169 |
| 5 | session 1 end | new | 0.944480 | 0.407546 |
| 6 | session 1 start | same | 0.934629 | 0.234032 |
| 7 | session 1 end | same | 0.968621 | 0.748577 |
| 8 | session 2 start | comp | 0.954532 | 0.500438 |
| 9 | session 2 end | comp | 0.962600 | 0.652508 |
| 10 | session 2 start | inco | 0.956258 | 0.594730 |
| 11 | session 2 end | inco | 0.961306 | 0.656151 |
| 12 | session 2 start | new | 0.945998 | 0.429055 |
| 13 | session 2 end | new | 0.930085 | 0.218357 |
| 14 | session 2 start | same | 0.929306 | 0.188805 |
| 15 | session 2 end | same | 0.964520 | 0.690605 |
| 16 | session 3 start | comp | 0.969510 | 0.809804 |
| 17 | session 3 end | comp | 0.937896 | 0.266645 |
| 18 | session 3 start | inco | 0.921244 | 0.155006 |
| 19 | session 3 end | inco | 0.852989 | 0.015081 |
| 20 | session 3 start | new | 0.950082 | 0.457823 |
| 21 | session 3 end | new | 0.963893 | 0.732616 |
| 22 | session 3 start | same | 0.962242 | 0.617255 |
| 23 | session 3 end | same | 0.956396 | 0.565031 |
| 24 | session 4 start | comp | 0.903130 | 0.090213 |
| 25 | session 4 end | comp | 0.975579 | 0.893484 |
| 26 | session 4 start | inco | 0.944893 | 0.413310 |
| 27 | session 4 end | inco | 0.951925 | 0.520782 |
| 28 | session 4 start | new | 0.920029 | 0.168861 |
| 29 | session 4 end | new | 0.932897 | 0.270859 |
| 30 | session 4 start | same | 0.936261 | 0.249836 |
| 31 | session 4 end | same | 0.955466 | 0.486745 |

**Supplementary Table 3.** Output of the repeated measures ANOVA and post hoc comparisons of the factor timepoint on the Δ compound values for the group Same. Mauchly's test of sphericity indicates that sphericity was violated ($p<.001$), thus, Greenhouse-Geisser Correction (GG) was applied.

**Within Subjects Effects (Group: Same)**

| Cases | Sphericity Correction | Sum of Squares | df | Mean Square | F | p |
|---|---|---|---|---|---|---|
| timepoint | GG | 0.881 | 2.893 | 0.304 | 56.566 | < .001 |
| Residuals | GG | 0.218 | 40.507 | 0.005 | | |

**Post Hoc Comparisons - timepoint (Group: Same)**

| | | Mean Difference | SE | t | $p_{bonf}$ |
|---|---|---|---|---|---|
| session 1 start | session 1 end | −0.095 | 0.017 | −5.581 | 0.002 |
| | session 2 start | −0.127 | 0.015 | −8.497 | < .001 |
| | session 2 end | −0.197 | 0.016 | −12.096 | < .001 |
| | session 3 start | −0.194 | 0.019 | −9.939 | < .001 |
| | session 3 end | −0.234 | 0.017 | −13.699 | < .001 |
| | session 4 start | −0.241 | 0.023 | −10.429 | < .001 |
| | session 4 end | −0.279 | 0.026 | −10.647 | < .001 |
| session 1 end | session 2 start | −0.032 | 0.017 | −1.884 | 1.000 |
| | session 2 end | −0.102 | 0.018 | −5.644 | 0.002 |
| | session 3 start | −0.099 | 0.019 | −5.321 | 0.003 |
| | session 3 end | −0.139 | 0.020 | −6.840 | < .001 |
| | session 4 start | −0.146 | 0.025 | −5.747 | 0.001 |
| | session 4 end | −0.184 | 0.026 | −6.964 | < .001 |
| session 2 start | session 2 end | −0.070 | 0.013 | −5.365 | 0.003 |
| | session 3 start | −0.067 | 0.013 | −5.269 | 0.003 |
| | session 3 end | −0.107 | 0.014 | −7.525 | < .001 |
| | session 4 start | −0.114 | 0.017 | −6.916 | < .001 |
| | session 4 end | −0.152 | 0.023 | −6.709 | < .001 |
| session 2 end | session 3 start | 0.003 | 0.008 | 0.390 | 1.000 |
| | session 3 end | −0.037 | 0.008 | −4.835 | 0.007 |
| | session 4 start | −0.044 | 0.015 | −3.022 | 0.256 |
| | session 4 end | −0.082 | 0.015 | −5.360 | 0.003 |
| session 3 start | session 3 end | −0.040 | 0.008 | −4.833 | 0.007 |
| | session 4 start | −0.047 | 0.011 | −4.503 | 0.014 |
| | session 4 end | −0.086 | 0.016 | −5.275 | 0.003 |
| session 3 end | session 4 start | −0.007 | 0.011 | −0.664 | 1.000 |
| | session 4 end | −0.046 | 0.012 | −3.654 | 0.073 |
| session 4 start | session 4 end | −0.038 | 0.017 | −2.217 | 1.000 |

**Supplementary Table 4.** Output of the repeated measures ANOVA and post hoc comparisons of the factor timepoint on the Δ compound values for the group New (in experiment 1, 4 days). Mauchly's test of sphericity indicates that sphericity was violated (p=.014), thus, Greenhouse-Geisser Correction (GG) was applied.

**Within Subjects Effects (Group: New)**

| Cases | Sphericity Correction | Sum of Squares | df | Mean Square | F | p |
|---|---|---|---|---|---|---|
| timepoint | GG | 0.339 | 2.861 | 0.119 | 16.840 | $< .001$ |
| Residuals | GG | 0.242 | 34.331 | 0.007 | | |

**Post Hoc Comparisons - timepoint (Group: New)**

| | | Mean Difference | SE | t | $p_{bonf}$ |
|---|---|---|---|---|---|
| session 1 start | session 1 end | −0.078 | 0.026 | −2.973 | 0.326 |
| | session 2 start | −0.104 | 0.026 | −3.972 | 0.052 |
| | session 2 end | −0.152 | 0.034 | −4.469 | 0.021 |
| | session 3 start | −0.142 | 0.033 | −4.283 | 0.030 |
| | session 3 end | −0.191 | 0.030 | −6.457 | $< .001$ |
| | session 4 start | −0.055 | 0.022 | −2.529 | 0.741 |
| | session 4 end | −0.135 | 0.032 | −4.224 | 0.033 |
| session 1 end | session 2 start | −0.026 | 0.012 | −2.222 | 1.000 |
| | session 2 end | −0.074 | 0.018 | −4.080 | 0.043 |
| | session 3 start | −0.064 | 0.019 | −3.401 | 0.147 |
| | session 3 end | −0.113 | 0.019 | −5.899 | 0.002 |
| | session 4 start | 0.023 | 0.015 | 1.499 | 1.000 |
| | session 4 end | −0.057 | 0.021 | −2.751 | 0.492 |
| session 2 start | session 2 end | −0.048 | 0.018 | −2.695 | 0.546 |
| | session 3 start | −0.037 | 0.017 | −2.212 | 1.000 |
| | session 3 end | −0.087 | 0.020 | −4.333 | 0.027 |
| | session 4 start | 0.049 | 0.016 | 3.151 | 0.234 |
| | session 4 end | −0.030 | 0.017 | −1.762 | 1.000 |
| session 2 end | session 3 start | 0.011 | 0.007 | 1.577 | 1.000 |
| | session 3 end | −0.039 | 0.017 | −2.212 | 1.000 |
| | session 4 start | 0.097 | 0.019 | 4.995 | 0.009 |
| | session 4 end | 0.018 | 0.014 | 1.296 | 1.000 |
| session 3 start | session 3 end | −0.049 | 0.015 | −3.316 | 0.172 |
| | session 4 start | 0.087 | 0.020 | 4.329 | 0.027 |
| | session 4 end | 0.007 | 0.013 | 0.562 | 1.000 |
| session 3 end | session 4 start | 0.136 | 0.023 | 5.959 | 0.002 |
| | session 4 end | 0.056 | 0.021 | 2.623 | 0.623 |
| session 4 start | session 4 end | −0.080 | 0.018 | −4.509 | 0.020 |

**Supplementary Table 5.** Output of the repeated measures ANOVA and post hoc comparisons of the factor timepoint on the Δ compound values for the group Compatible (in experiment 1, 4 days). Mauchly's test of sphericity indicates that sphericity was violated (p=.002), thus, Greenhouse-Geisser Correction (GG) was applied.

**Within Subjects Effects (Group: Compatible)**

| Cases | Sphericity Correction | Sum of Squares | df | Mean Square | F | p |
|---|---|---|---|---|---|---|
| timepoint | GG | 0.580 | 3.577 | 0.162 | 54.772 | $< .001$ |
| Residuals | GG | 0.148 | 50.072 | 0.003 | | |

**Post Hoc Comparisons - timepoint (Group: Compatible)**

| | | Mean Difference | SE | t | $p_{bonf}$ |
|---|---|---|---|---|---|
| session 1 start | session 1 end | −0.122 | 0.023 | −5.354 | 0.003 |
| | session 2 start | −0.108 | 0.014 | −7.835 | $< .001$ |
| | session 2 end | −0.182 | 0.013 | −13.622 | $< .001$ |
| | session 3 start | −0.179 | 0.011 | −15.587 | $< .001$ |
| | session 3 end | −0.232 | 0.016 | −14.678 | $< .001$ |
| | session 4 start | −0.129 | 0.012 | −10.415 | $< .001$ |
| | session 4 end | −0.219 | 0.015 | −14.759 | $< .001$ |
| session 1 end | session 2 start | 0.014 | 0.019 | 0.756 | 1.000 |
| | session 2 end | −0.060 | 0.015 | −4.142 | 0.028 |
| | session 3 start | −0.057 | 0.019 | −3.069 | 0.233 |
| | session 3 end | −0.110 | 0.020 | −5.584 | 0.002 |
| | session 4 start | −0.007 | 0.017 | −0.393 | 1.000 |
| | session 4 end | −0.096 | 0.021 | −4.599 | 0.012 |
| session 2 start | session 2 end | −0.074 | 0.014 | −5.349 | 0.003 |
| | session 3 start | −0.071 | 0.013 | −5.650 | 0.002 |
| | session 3 end | −0.124 | 0.015 | −7.984 | $< .001$ |
| | session 4 start | −0.021 | 0.011 | −1.882 | 1.000 |
| | session 4 end | −0.110 | 0.016 | −7.094 | $< .001$ |
| session 2 end | session 3 start | 0.003 | 0.008 | 0.439 | 1.000 |
| | session 3 end | −0.049 | 0.010 | −4.836 | 0.007 |
| | session 4 start | 0.053 | 0.010 | 5.407 | 0.003 |
| | session 4 end | −0.036 | 0.014 | −2.555 | 0.641 |
| session 3 start | session 3 end | −0.053 | 0.011 | −4.680 | 0.010 |
| | session 4 start | 0.050 | 0.008 | 6.192 | $< .001$ |
| | session 4 end | −0.039 | 0.010 | −3.794 | 0.055 |
| session 3 end | session 4 start | 0.103 | 0.009 | 11.366 | $< .001$ |
| | session 4 end | 0.013 | 0.011 | 1.225 | 1.000 |
| session 4 start | session 4 end | −0.089 | 0.008 | −11.723 | $< .001$ |

**Supplementary Table 6.** Output of the repeated measures ANOVA and post hoc comparisons of the factor timepoint on the Δ compound values for the group Incompatible (in experiment 1 - 4 days). Mauchly's test of sphericity: p=.212

**Within Subjects Effects (Group: Incompatible)**

| Cases | Sphericity Correction | Sum of Squares | df | Mean Square | F | p |
|---|---|---|---|---|---|---|
| timepoint | None | 0.620 | 7.000 | 0.089 | 30.290 | $< .001$ |
| Residuals | None | 0.246 | 84.000 | 0.003 | | |

**Post Hoc Comparisons - timepoint (Group: Incompatible)**

| | | Mean Difference | SE | t | $p_{bonf}$ |
|---|---|---|---|---|---|
| session 1 start | session 1 end | −0.144 | 0.019 | −7.742 | $< .001$ |
| | session 2 start | −0.137 | 0.020 | −6.734 | $< .001$ |
| | session 2 end | −0.195 | 0.019 | −10.031 | $< .001$ |
| | session 3 start | −0.205 | 0.028 | −7.386 | $< .001$ |
| | session 3 end | −0.240 | 0.023 | −10.408 | $< .001$ |
| | session 4 start | −0.150 | 0.017 | −8.555 | $< .001$ |
| | session 4 end | −0.270 | 0.031 | −8.568 | $< .001$ |
| session 1 end | session 2 start | 0.007 | 0.015 | 0.459 | 1.000 |
| | session 2 end | −0.052 | 0.021 | −2.503 | 0.778 |
| | session 3 start | −0.062 | 0.023 | −2.719 | 0.522 |
| | session 3 end | −0.096 | 0.021 | −4.640 | 0.016 |
| | session 4 start | −0.006 | 0.019 | −0.320 | 1.000 |
| | session 4 end | −0.126 | 0.029 | −4.348 | 0.027 |
| session 2 start | session 2 end | −0.059 | 0.019 | −3.073 | 0.270 |
| | session 3 start | −0.069 | 0.018 | −3.900 | 0.059 |
| | session 3 end | −0.103 | 0.019 | −5.490 | 0.004 |
| | session 4 start | −0.013 | 0.021 | −0.614 | 1.000 |
| | session 4 end | −0.133 | 0.019 | −6.930 | $< .001$ |
| session 2 end | session 3 start | −0.010 | 0.018 | −0.547 | 1.000 |
| | session 3 end | −0.044 | 0.012 | −3.561 | 0.110 |
| | session 4 start | 0.046 | 0.012 | 3.932 | 0.056 |
| | session 4 end | −0.074 | 0.027 | −2.724 | 0.518 |
| session 3 start | session 3 end | −0.034 | 0.015 | −2.273 | 1.000 |
| | session 4 start | 0.056 | 0.020 | 2.803 | 0.447 |
| | session 4 end | −0.064 | 0.023 | −2.797 | 0.452 |
| session 3 end | session 4 start | 0.090 | 0.015 | 6.127 | 0.001 |
| | session 4 end | −0.030 | 0.025 | −1.190 | 1.000 |
| session 4 start | session 4 end | −0.120 | 0.029 | −4.120 | 0.040 |

Since the Δ compound values for the group Incompatible showed a non-normal distribution at the end of session 3 (see Table S2), we also performed the Friedman test as a non-parametric alternative to the ANOVA, using Conover's test for post hoc comparisons (results are reported in Table S7).

**Supplementary Table 7.** Output of the non-parametric Friedman test and post hoc comparisons of the factor timepoint on the Δ compound values for the group Incompatible.

**Friedman Test (Group: Incompatible)**

| Factor | $X^2_F$ | df | p | Kendall's W |
|---|---|---|---|---|
| timepoint | 67.718 | 7 | $< .001$ | 0.744 |

**Conover's Post Hoc Comparisons - timepoint (Group: Incompatible)**

| | | T-Stat | df | $W_i$ | $W_j$ | $r_{rb}$ | p | $p_{bonf}$ | $p_{holm}$ |
|---|---|---|---|---|---|---|---|---|---|
| session 1 start | session 1 end | 5.323 | 84 | 14.000 | 49.000 | −1.000 | < .001 | < .001 | < .001 |
| | session 2 start | 3.498 | 84 | 14.000 | 37.000 | −1.000 | < .001 | 0.021 | 0.008 |
| | session 2 end | 7.908 | 84 | 14.000 | 66.000 | −0.978 | < .001 | < .001 | < .001 |
| | session 3 start | 9.733 | 84 | 14.000 | 78.000 | −1.000 | < .001 | < .001 | < .001 |
| | session 3 end | 11.102 | 84 | 14.000 | 87.000 | −1.000 | < .001 | < .001 | < .001 |
| | session 4 start | 4.410 | 84 | 14.000 | 43.000 | −1.000 | < .001 | < .001 | < .001 |
| | session 4 end | 12.166 | 84 | 14.000 | 94.000 | −1.000 | < .001 | < .001 | < .001 |
| session 1 end | session 2 start | 1.825 | 84 | 49.000 | 37.000 | 0.253 | 0.072 | 1.000 | 0.429 |
| | session 2 end | 2.585 | 84 | 49.000 | 66.000 | −0.670 | 0.011 | 0.321 | 0.092 |
| | session 3 start | 4.410 | 84 | 49.000 | 78.000 | −0.714 | < .001 | < .001 | < .001 |
| | session 3 end | 5.779 | 84 | 49.000 | 87.000 | −0.978 | < .001 | < .001 | < .001 |
| | session 4 start | 0.912 | 84 | 49.000 | 43.000 | 0.011 | 0.364 | 1.000 | 0.870 |
| | session 4 end | 6.844 | 84 | 49.000 | 94.000 | −1.000 | < .001 | < .001 | < .001 |
| session 2 start | session 2 end | 4.410 | 84 | 37.000 | 66.000 | −0.736 | < .001 | < .001 | < .001 |
| | session 3 start | 6.235 | 84 | 37.000 | 78.000 | −1.000 | < .001 | < .001 | < .001 |
| | session 3 end | 7.604 | 84 | 37.000 | 87.000 | −0.978 | < .001 | < .001 | < .001 |
| | session 4 start | 0.912 | 84 | 37.000 | 43.000 | −0.231 | 0.364 | 1.000 | 0.870 |
| | session 4 end | 8.668 | 84 | 37.000 | 94.000 | −1.000 | < .001 | < .001 | < .001 |
| session 2 end | session 3 start | 1.825 | 84 | 66.000 | 78.000 | −0.341 | 0.072 | 1.000 | 0.429 |
| | session 3 end | 3.194 | 84 | 66.000 | 87.000 | −0.846 | 0.002 | 0.055 | 0.018 |
| | session 4 start | 3.498 | 84 | 66.000 | 43.000 | 0.868 | < .001 | 0.021 | 0.008 |
| | session 4 end | 4.258 | 84 | 66.000 | 94.000 | −0.802 | < .001 | 0.001 | < .001 |
| session 3 start | session 3 end | 1.369 | 84 | 78.000 | 87.000 | −0.582 | 0.175 | 1.000 | 0.699 |
| | session 4 start | 5.323 | 84 | 78.000 | 43.000 | 0.714 | < .001 | < .001 | < .001 |
| | session 4 end | 2.433 | 84 | 78.000 | 94.000 | −0.736 | 0.017 | 0.478 | 0.120 |
| session 3 end | session 4 start | 6.691 | 84 | 87.000 | 43.000 | 1.000 | < .001 | < .001 | < .001 |
| | session 4 end | 1.065 | 84 | 87.000 | 94.000 | −0.275 | 0.290 | 1.000 | 0.870 |
| session 4 start | session 4 end | 7.756 | 84 | 43.000 | 94.000 | −1.000 | < .001 | < .001 | < .001 |

## C. Statistical analysis - $\Delta$ RT in novel versus learned transitions

**Supplementary Table 8. Output of the normality tests (Shapiro-Wilk test) of the $\Delta$ RT for the novel and learned transitions in each group in session 4 (start and end of session 4).** The analysis was performed in Python using the scipy package [2].

| | timepoint | condition | group | Shapiro-Wilk test statistic | p-value |
|---|---|---|---|---|---|
| 0 | session 4 start | learned | comp | 0.923280 | 0.147771 |
| 1 | session 4 end | learned | comp | 0.972001 | 0.834369 |
| 2 | session 4 start | learned | inco | 0.933089 | 0.303320 |
| 3 | session 4 end | learned | inco | 0.981818 | 0.980397 |
| 6 | session 4 start | learned | same | 0.920060 | 0.129590 |
| 7 | session 4 end | learned | same | 0.951790 | 0.423649 |
| 8 | session 4 start | novel | comp | 0.962482 | 0.678495 |
| 9 | session 4 end | novel | comp | 0.943292 | 0.359336 |
| 10 | session 4 start | novel | inco | 0.919793 | 0.167394 |
| 11 | session 4 end | novel | inco | 0.950181 | 0.459393 |
| 12 | session 4 start | novel | new | 0.921903 | 0.180999 |
| 13 | session 4 end | novel | new | 0.954611 | 0.566053 |

## 2. RESULTS: EXPERIMENT 2 (2 DAYS)

### A. Performance measures: reaction times and accuracy

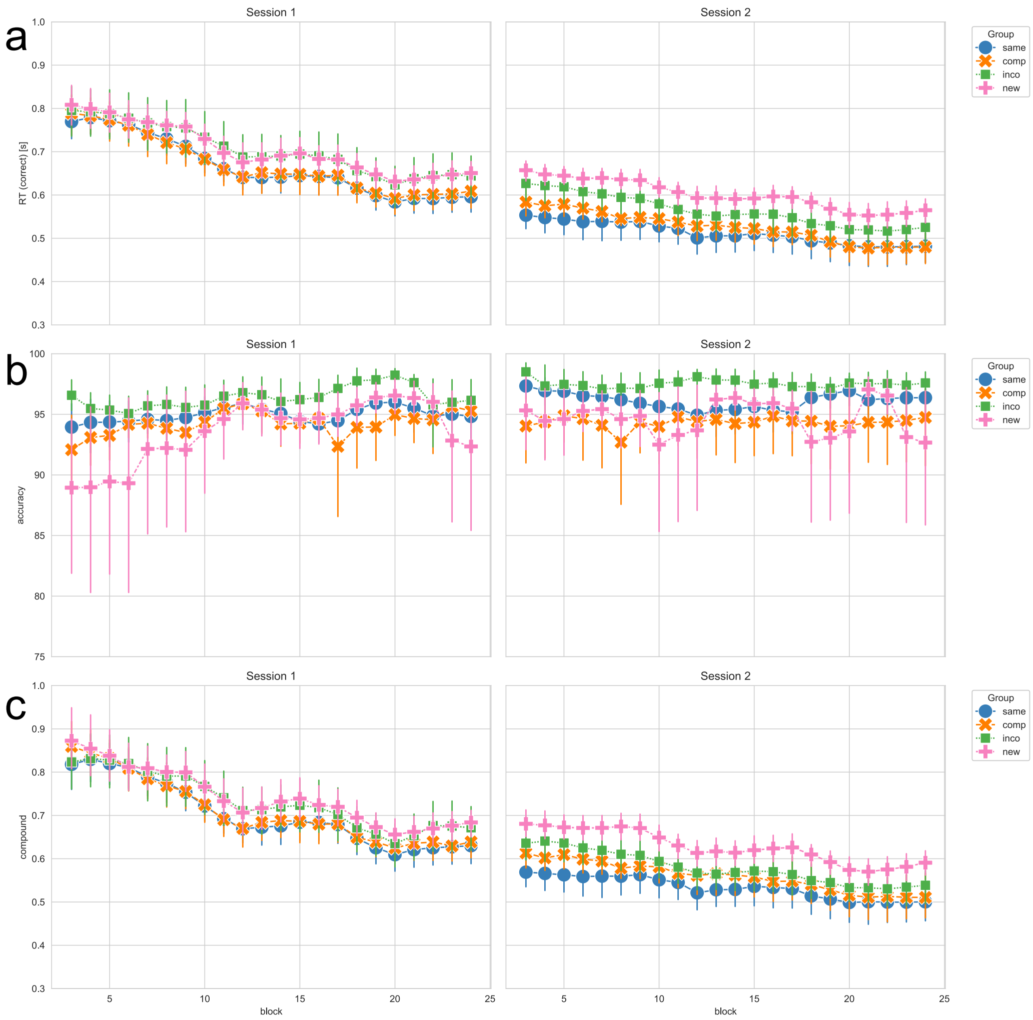


**Supplementary Figure 3.** Performance measures in the SEQUENCE condition in experiment 2 across the 2 training sessions and experimental groups. a) shows the reaction time (RT) for correctly executed sequences; b) shows the accuracy in terms of the number of correct key presses divided by the number of sequence elements (8); c) shows the compound measure which is based on the RT of correct key presses scaled by the accuracy/number of errors, thus combining RT and accuracy. The experimental groups "same", "comp" (=compatible), "inco" (=incompatible), and "new" are color-coded in blue, orange, green, and pink, respectively. While each row shows a specific behavioral measure (RT, accuracy, compound measure), each column shows a training session. Data is depicted using a sliding window approach (moving average smoothing, n=3). Vertical bars represent the 95 % confidence intervals.

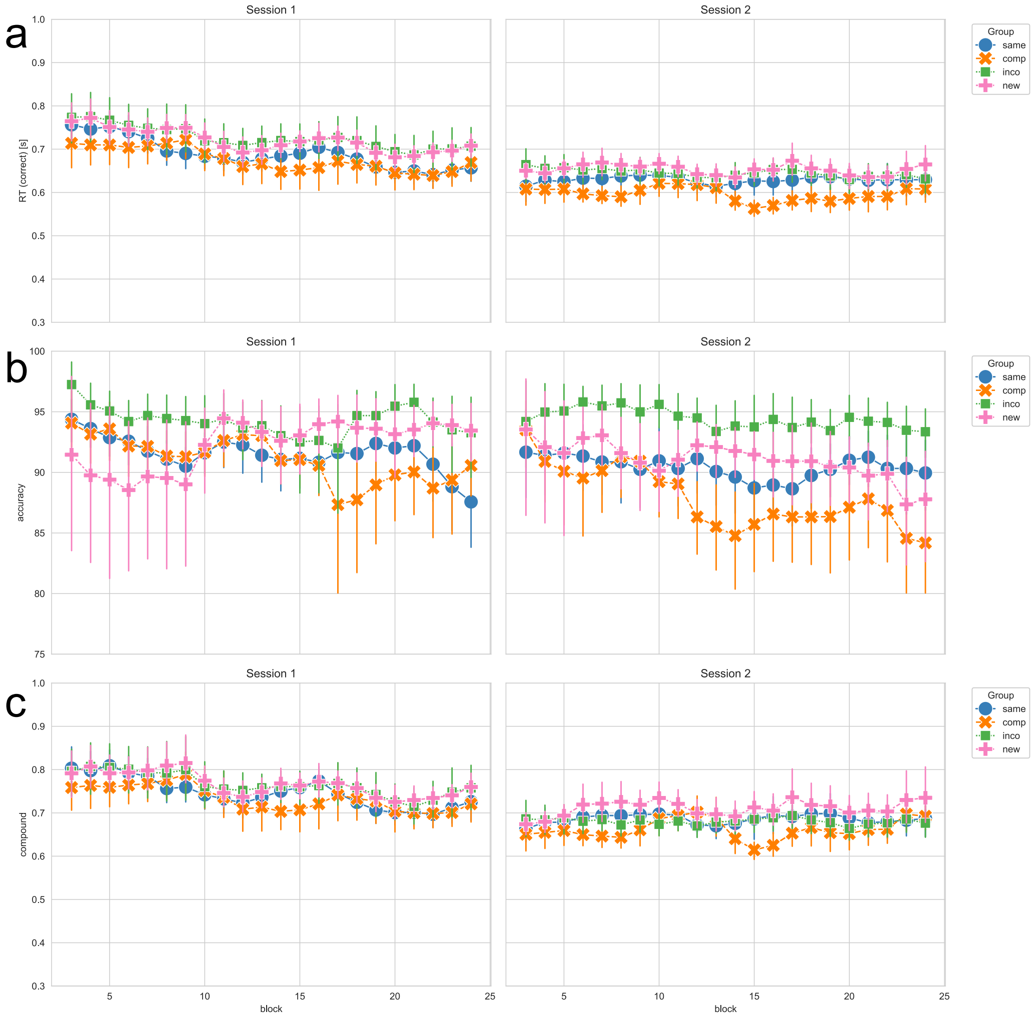


**Supplementary Figure 4.** Performance measures in the RANDOM condition in experiment 2 across the 2 training sessions and experimental groups. a) shows the reaction time (RT) for correctly executed sequences; b) shows the accuracy in terms of the number of correct key presses divided by the number of sequence elements (8); c) shows the compound measure which is based on the RT of correct key presses scaled by the accuracy/number of errors, thus combining RT and accuracy. The experimental groups "same", "comp" (=compatible), "inco" (=incompatible), and "new" are color-coded in blue, orange, green, and pink, respectively. While each row shows a specific behavioral measure (RT, accuracy, compound measure), each column shows a training session. Data is depicted using a sliding window approach (moving average smoothing, n=3). Vertical bars represent the 95 % confidence intervals.

## B. Statistical analysis - Δ compound measure

**Supplementary Table 9. Output of the mixed ANOVA of the Δ compound values with the timepoint as a within subject factor and the experimental group as a between subject factor.** The timepoints in experiment 2 consisted of: session 1 start, session 1 end, session 2 start, session 2 end. A sphericity test revealed that sphericity was met (p=.051). The analysis was performed in JASP [1].

| Within Subjects Effects | | | | | |
|---|---|---|---|---|---|
| Cases | Sum of Squares | df | Mean Square | F | p |
| timepoint | 0.980 | 3 | 0.327 | 174.331 | $< .001$ |
| timepoint * group | 0.076 | 9 | 0.008 | 4.484 | $< .001$ |
| Residuals | 0.298 | 159 | 0.002 | | |
| **Between Subjects Effects** | | | | | |
| Cases | Sum of Squares | df | Mean Square | F | p |
| group | 0.054 | 3 | 0.018 | 4.918 | 0.004 |
| Residuals | 0.195 | 53 | 0.004 | | |

**Supplementary Table 10. Output of the normality tests (Shapiro-Wilk test) of the Δ compound values for each group and each timepoint.** The timepoints in experiment 2 consisted of: session 1 start, session 1 end, session 2 start, session 2 end. The analysis was performed in Python using the scipy package [2].

| | timepoint | group | Shapiro-Wilk Test Statistic | p-value |
|---|---|---|---|---|
| 0 | session 1 start | comp | 0.864196 | 0.011519 |
| 1 | session 1 end | comp | 0.920513 | 0.150652 |
| 2 | session 1 start | inco | 0.880261 | 0.032108 |
| 3 | session 1 end | inco | 0.852569 | 0.009299 |
| 4 | session 1 start | new | 0.890615 | 0.068511 |
| 5 | session 1 end | new | 0.951500 | 0.513812 |
| 6 | session 1 start | same | 0.918141 | 0.104560 |
| 7 | session 1 end | same | 0.968621 | 0.748577 |
| 8 | session 2 start | comp | 0.874621 | 0.026041 |
| 9 | session 2 end | comp | 0.963762 | 0.730231 |
| 10 | session 2 start | inco | 0.936784 | 0.282000 |
| 11 | session 2 end | inco | 0.956293 | 0.563185 |
| 12 | session 2 start | new | 0.827220 | 0.006410 |
| 13 | session 2 end | new | 0.699750 | 0.000168 |
| 14 | session 2 start | same | 0.918728 | 0.107203 |
| 15 | session 2 end | same | 0.964520 | 0.690605 |

**Supplementary Table 11.** Output of the repeated measures ANOVA and post hoc comparisons of the factor timepoint on the Δ compound values for the group Same. Mauchly's test of sphericity indicates that sphericity was met (p<.920).

**Within Subjects Effects (Group: Same)**

| Cases | Sphericity Correction | Sum of Squares | df | Mean Square | F | p |
|---|---|---|---|---|---|---|
| timepoint | None | 0.339 | 3.000 | 0.113 | 54.404 | $< .001$ |
| Residuals | None | 0.094 | 45.000 | 0.002 | | |

**Post Hoc Comparisons - timepoint (Group: Same)**

| | | Mean Difference | SE | t | $p_{bonf}$ |
|---|---|---|---|---|---|
| session 1 start | session 1 end | −0.098 | 0.016 | −6.041 | $< .001$ |
| | session 2 start | −0.137 | 0.017 | −8.046 | $< .001$ |
| | session 2 end | −0.201 | 0.016 | −12.768 | $< .001$ |
| session 1 end | session 2 start | −0.038 | 0.017 | −2.238 | 0.245 |
| | session 2 end | −0.102 | 0.017 | −6.056 | $< .001$ |
| session 2 start | session 2 end | −0.064 | 0.013 | −4.778 | 0.001 |

**Supplementary Table 12.** Output of the repeated measures ANOVA and post hoc comparisons of the factor timepoint on the Δ compound values for the group Compatible. Mauchly's test of sphericity indicates that sphericity was violated ($p<.001$), thus Greenhouse-Geisser correction (GG) was applied.

**Within Subjects Effects (Group: Compatible)**

| Cases | Sphericity Correction | Sum of Squares | df | Mean Square | F | p |
|---|---|---|---|---|---|---|
| timepoint | GG | 0.403 | 1.842 | 0.219 | 56.605 | < .001 |
| Residuals | GG | 0.092 | 23.942 | 0.004 | | |

**Post Hoc Comparisons - timepoint (Group: Compatible)**

| | | Mean Difference | SE | t | $p_{bonf}$ |
|---|---|---|---|---|---|
| session 1 start | session 1 end | −0.135 | 0.018 | −7.711 | < .001 |
| | session 2 start | −0.115 | 0.016 | −7.264 | < .001 |
| | session 2 end | −0.239 | 0.026 | −9.337 | < .001 |
| session 1 end | session 2 start | 0.021 | 0.005 | 4.317 | 0.005 |
| | session 2 end | −0.103 | 0.020 | −5.204 | 0.001 |
| session 2 start | session 2 end | −0.124 | 0.020 | −6.180 | < .001 |

Since the Δ compound values for the group Compatible showed a non-normal distribution at the start of session 1 and the start of session 2 (see Table S10), we also performed the Friedman test as a non-parametric alternative to the ANOVA, using Conover's test for post hoc comparisons (results are reported in Table S13).

**Supplementary Table 13.** Output of the non-parametric Friedman test and post hoc comparisons of the factor timepoint on the Δ compound values for the group Compatible.

**Friedman Test (Group: Compatible)**

| Factor | $X^2_F$ | df | p | Kendall's W |
|---|---|---|---|---|
| timepoint | 39.171 | 3 | < .001 | 0.933 |

**Conover's Post Hoc Comparisons - timepoint (Group: Compatible)**

| | | T-Stat | df | $W_i$ | $W_j$ | $r_{rb}$ | p | $p_{bonf}$ | $p_{holm}$ |
|---|---|---|---|---|---|---|---|---|---|
| session 1 start | session 1 end | 13.589 | 39 | 14.000 | 39.000 | −1.000 | < .001 | < .001 | < .001 |
| | session 2 start | 9.240 | 39 | 14.000 | 31.000 | −1.000 | < .001 | < .001 | < .001 |
| | session 2 end | 22.829 | 39 | 14.000 | 56.000 | −1.000 | < .001 | < .001 | < .001 |
| session 1 end | session 2 start | 4.348 | 39 | 39.000 | 31.000 | 0.886 | < .001 | < .001 | < .001 |
| | session 2 end | 9.240 | 39 | 39.000 | 56.000 | −1.000 | < .001 | < .001 | < .001 |
| session 2 start | session 2 end | 13.589 | 39 | 31.000 | 56.000 | −1.000 | < .001 | < .001 | < .001 |

**Supplementary Table 14.** Output of the repeated measures ANOVA and post hoc comparisons of the factor timepoint on the Δ compound values for the group Incompatible. Mauchly's test of sphericity indicates that sphericity was met (p<.436).

**Within Subjects Effects (Group: Incompatible)**

| Cases | Sphericity Correction | Sum of Squares | df | Mean Square | F | p |
|---|---|---|---|---|---|---|
| timepoint | None | 0.221 | 3.000 | 0.074 | 53.112 | $< .001$ |
| Residuals | None | 0.058 | 42.000 | 0.001 | | |

**Post Hoc Comparisons - timepoint (Group: Incompatible)**

| | | Mean Difference | SE | t | $p_{bonf}$ |
|---|---|---|---|---|---|
| session 1 start | session 1 end | −0.090 | 0.013 | −7.075 | $< .001$ |
| | session 2 start | −0.071 | 0.012 | −6.033 | $< .001$ |
| | session 2 end | −0.170 | 0.016 | −10.387 | $< .001$ |
| session 1 end | session 2 start | 0.019 | 0.013 | 1.523 | 0.901 |
| | session 2 end | −0.080 | 0.016 | −5.043 | 0.001 |
| session 2 start | session 2 end | −0.100 | 0.011 | −8.883 | $< .001$ |

Since the Δ compound measures showed a non-normal distribution in session 1 for the Incompatible group (see Table S10), we also performed non-parametric alternatives to the ANOVA and post hoc comparisons, reported in Table S15.

**Supplementary Table 15.** Output of the non-parametric Friedman test and post hoc comparisons of the factor timepoint on the Δ compound values for the group Incompatible.

**Friedman Test (Group: Incompatible)**

| Factor | $X^2{}_F$ | df | p | Kendall's W |
|---|---|---|---|---|
| timepoint | 37.160 | 3 | $< .001$ | 0.826 |

**Conover's Post Hoc Comparisons - timepoint (Group: Incompatible)**

| | | T-Stat | df | $W_i$ | $W_j$ | $r_{rb}$ | p | $p_{bonf}$ | $p_{holm}$ |
|---|---|---|---|---|---|---|---|---|---|
| session 1 start | session 1 end | 7.529 | 42 | 16.000 | 39.000 | −1.000 | $< .001$ | $< .001$ | $< .001$ |
| | session 2 start | 6.547 | 42 | 16.000 | 36.000 | −0.967 | $< .001$ | $< .001$ | $< .001$ |
| | session 2 end | 14.075 | 42 | 16.000 | 59.000 | −1.000 | $< .001$ | $< .001$ | $< .001$ |
| session 1 end | session 2 start | 0.982 | 42 | 39.000 | 36.000 | 0.333 | 0.332 | 1.000 | 0.332 |
| | session 2 end | 6.547 | 42 | 39.000 | 59.000 | −0.967 | $< .001$ | $< .001$ | $< .001$ |
| session 2 start | session 2 end | 7.529 | 42 | 36.000 | 59.000 | −1.000 | $< .001$ | $< .001$ | $< .001$ |

**Supplementary Table 16.** Output of the repeated measures ANOVA and post hoc comparisons of the factor timepoint on the Δ compound values for the group New. Mauchly's test of sphericity indicates that sphericity was met (p<.598).

**Within Subjects Effects (Group: New)**

| Cases | Sphericity Correction | Sum of Squares | df | Mean Square | F | p |
|---|---|---|---|---|---|---|
| timepoint | None | 0.125 | 3.000 | 0.042 | 25.524 | $< .001$ |
| Residuals | None | 0.054 | 33.000 | 0.002 | | |

**Post Hoc Comparisons - timepoint (Group: New)**

| | | Mean Difference | SE | t | $p_{bonf}$ |
|---|---|---|---|---|---|
| session 1 start | session 1 end | −0.102 | 0.018 | −5.669 | $< .001$ |
| | session 2 start | −0.047 | 0.016 | −2.986 | 0.074 |
| | session 2 end | −0.133 | 0.020 | −6.539 | $< .001$ |
| session 1 end | session 2 start | 0.055 | 0.011 | 4.868 | 0.003 |
| | session 2 end | −0.031 | 0.016 | −1.928 | 0.481 |
| session 2 start | session 2 end | −0.086 | 0.016 | −5.299 | 0.002 |

Since the Δ compound measures in the New group were not normally distributed in session 2 (see Table S10), we also performed non-parametric alternatives to the ANOVA and post hoc comparisons (reported in Table S17).

**Supplementary Table 17.** Output of the non-parametric Friedman test and post hoc comparisons of the factor timepoint on the Δ compound values for the group New.

**Friedman Test (Group: New)**

| Factor | $X^2{}_F$ | df | p | Kendall's W |
|---|---|---|---|---|
| timepoint | 26.700 | 3 | $< .001$ | 0.742 |

**Conover's Post Hoc Comparisons - timepoint (Group: New)**

| | | T-Stat | df | $W_i$ | $W_j$ | $r_{rb}$ | p | $p_{bonf}$ | $p_{holm}$ |
|---|---|---|---|---|---|---|---|---|---|
| session 1 start | session 1 end | 6.850 | 33 | 15.000 | 38.000 | −0.974 | $< .001$ | $< .001$ | $< .001$ |
| | session 2 start | 2.383 | 33 | 15.000 | 23.000 | −0.795 | 0.023 | 0.139 | 0.046 |
| | session 2 end | 8.637 | 33 | 15.000 | 44.000 | −1.000 | $< .001$ | $< .001$ | $< .001$ |
| session 1 end | session 2 start | 4.468 | 33 | 38.000 | 23.000 | 0.897 | $< .001$ | $< .001$ | $< .001$ |
| | session 2 end | 1.787 | 33 | 38.000 | 44.000 | −0.538 | 0.083 | 0.499 | 0.083 |
| session 2 start | session 2 end | 6.255 | 33 | 23.000 | 44.000 | −1.000 | $< .001$ | $< .001$ | $< .001$ |

**Supplementary Table 18.** Output of the ANOVA and post hoc comparisons of the (between-subject) factor group on the Δ compound values, at the start of session 1.

**ANOVA (session 1 start)**

| Cases | Sum of Squares | df | Mean Square | F | p |
|---|---|---|---|---|---|
| group | 0.024 | 3 | 0.008 | 3.614 | 0.018 |
| Residuals | 0.135 | 62 | 0.002 | | |

**Post Hoc Comparisons - group (session 1 start)**

| | | Mean Difference | SE | t | $p_{bonf}$ |
|---|---|---|---|---|---|
| comp | inco | −0.052 | 0.016 | −3.228 | 0.012 |
| | new | −0.033 | 0.017 | −2.015 | 0.289 |
| | same | −0.029 | 0.016 | −1.896 | 0.376 |
| inco | new | 0.018 | 0.017 | 1.069 | 1.000 |
| | same | 0.022 | 0.016 | 1.389 | 1.000 |
| new | same | 0.004 | 0.017 | 0.242 | 1.000 |

The Δ compound measures in the Compatible and Incompatible group were not normally distributed at the start of session 1 (see Table S10), thus, we also performed non-parametric alternatives to the ANOVA and post hoc comparisons, reported in Table S19).

**Supplementary Table 19.** Output of the non-parametric Kruskal-Wallis test and Dunn's post hoc comparisons of the (between-subject) factor group on the Δ compound values, at the start of session 1.

**Kruskal-Wallis Test (session 1 start)**

| Factor | Statistic | df | p |
|---|---|---|---|
| group | 11.331 | 3 | 0.010 |

**Dunn's Post Hoc Comparisons - group (session 1 start)**

| Comparison | z | $W_i$ | $W_j$ | $r_{rb}$ | p | $p_{bonf}$ | $p_{holm}$ |
|---|---|---|---|---|---|---|---|
| comp - inco | −3.307 | 22.500 | 44.313 | 0.639 | < .001 | 0.006 | 0.006 |
| comp - new | −2.026 | 22.500 | 36.357 | 0.421 | 0.043 | 0.257 | 0.214 |
| comp - same | −1.589 | 22.500 | 32.667 | 0.327 | 0.112 | 0.673 | 0.336 |
| inco - new | 1.132 | 44.313 | 36.357 | 0.232 | 0.257 | 1.000 | 0.515 |
| inco - same | 1.766 | 44.313 | 32.667 | 0.382 | 0.077 | 0.465 | 0.310 |
| new - same | 0.539 | 36.357 | 32.667 | 0.103 | 0.590 | 1.000 | 0.590 |

**Supplementary Table 20.** Output of the ANOVA of the (between-subject) factor group on the Δ compound values, at the end of session 1. Welch's ANOVA was used for homogeneity correction since the equality of variances assumption was violated, as probed by Levene's test (p=.002). Moreover, since the Δ compound values of the Incompatible group were not normally distributed (see Table S10), we also performed the non-parametric alternative: Kruskal-Wallis Test.

**ANOVA (session 1 end)**

| Homogeneity Correction | Cases | Sum of Squares | df | Mean Square | F | p |
|---|---|---|---|---|---|---|
| Welch | group | 0.011 | 3.000 | 0.004 | 1.073 | 0.374 |
| | Residuals | 0.182 | 33.515 | 0.005 | | |

**Kruskal-Wallis Test (session 1 end)**

| Factor | Statistic | df | p |
|---|---|---|---|
| group | 1.705 | 3 | 0.636 |

**Supplementary Table 21.** Output of the ANOVA of the (between-subject) factor group and post hoc tests on the Δ compound values, at the start of session 2. Welch's ANOVA was used for homogeneity correction since the equality of variances assumption was violated, as probed by Levene's test (p=.016).

**ANOVA (session 2 start)**

| Homogeneity Correction | Cases | Sum of Squares | df | Mean Square | F | p |
|---|---|---|---|---|---|---|
| Welch | group | 0.080 | 3.000 | 0.027 | 14.178 | $< .001$ |
| | Residuals | 0.102 | 33.232 | 0.003 | | |

**Post Hoc Comparisons - group (session 2 start)**

| | | Mean Difference | SE | t | $p_{bonf}$ |
|---|---|---|---|---|---|
| comp | inco | −0.003 | 0.014 | −0.193 | 1.000 |
| | new | 0.039 | 0.015 | 2.687 | 0.056 |
| | same | −0.058 | 0.014 | −4.134 | $< .001$ |
| inco | new | 0.042 | 0.015 | 2.877 | 0.033 |
| | same | −0.055 | 0.014 | −3.935 | 0.001 |
| new | same | −0.097 | 0.014 | −6.825 | $< .001$ |

Since the Δ compound measures in the Compatible and New group were not normally distributed at the start of session 1 (see Table S10), we also performed the Kruskal-Wallis test and Dunn's posthoc comparisons as non-parametric alternatives to the ANOVA - the results are reported in Table S22.

**Supplementary Table 22.** Output of the non-parametric Kruskal-Wallis test and Dunn's post hoc comparisons of the (between-subject) factor group on the Δ compound values, at the start of session 2.

**Kruskal-Wallis Test (session 2 start)**

| Factor | Statistic | df | p |
|---|---|---|---|
| group | 28.053 | 3 | $< .001$ |

**Dunn's Post Hoc Comparisons - group (session 2 start)**

| Comparison | z | $W_i$ | $W_j$ | $r_{rb}$ | p | $p_{bonf}$ | $p_{holm}$ |
|---|---|---|---|---|---|---|---|
| comp - inco | −0.037 | 31.875 | 32.125 | 0.039 | 0.970 | 1.000 | 0.970 |
| comp - new | 2.493 | 31.875 | 14.933 | 0.650 | 0.013 | 0.076 | 0.034 |
| comp - same | −2.764 | 31.875 | 49.833 | 0.701 | 0.006 | 0.034 | 0.029 |
| inco - new | 2.530 | 32.125 | 14.933 | 0.558 | 0.011 | 0.068 | 0.034 |
| inco - same | −2.726 | 32.125 | 49.833 | 0.528 | 0.006 | 0.038 | 0.029 |
| new - same | −5.280 | 14.933 | 49.833 | 0.933 | $< .001$ | $< .001$ | $< .001$ |

**Supplementary Table 23.** Output of the ANOVA of the (between-subject) factor group and post hoc tests on the Δ compound values, at the end of session 2. The equality of variances assumption was met, as probed by Levene's test (p=.267).

**ANOVA (session 2 end)**

| Cases | Sum of Squares | df | Mean Square | F | p |
|---|---|---|---|---|---|
| group | 0.056 | 3 | 0.019 | 4.735 | 0.005 |
| Residuals | 0.240 | 61 | 0.004 | | |

**Post Hoc Comparisons - group (session 2 end)**

| | | Mean Difference | SE | t | $p_{bonf}$ |
|---|---|---|---|---|---|
| comp | inco | 0.015 | 0.022 | 0.674 | 1.000 |
| | new | 0.073 | 0.023 | 3.176 | 0.014 |
| | same | $-7.453 \times 10^{-4}$ | 0.022 | −0.034 | 1.000 |
| inco | new | 0.058 | 0.022 | 2.600 | 0.070 |
| | same | −0.016 | 0.021 | −0.741 | 1.000 |
| new | same | −0.074 | 0.022 | −3.351 | 0.008 |

According to the Shapiro-Wilk test (see Table S10), the Δ compound measures in the New group were not normally distributed at the end of session 2, thus we also performed the Kruskal-Wallis test with Dunn's post hoc tests (see Table S24).

**Supplementary Table 24.** Output of the non-parametric Kruskal-Wallis test and Dunn's post hoc comparisons of the (between-subject) factor group on the Δ compound values, at the end of session 2.

**Kruskal-Wallis Test (session 2 end)**

| Factor | Statistic | df | p |
|---|---|---|---|
| group | 11.827 | 3 | 0.008 |

**Dunn's Post Hoc Comparisons - group (session 2 end)**

| Comparison | z | $W_i$ | $W_j$ | $r_{rb}$ | p | $p_{bonf}$ | $p_{holm}$ |
|---|---|---|---|---|---|---|---|
| comp - inco | 0.527 | 38.000 | 34.471 | 0.098 | 0.598 | 1.000 | 1.000 |
| comp - new | 2.800 | 38.000 | 18.667 | 0.556 | 0.005 | 0.031 | 0.026 |
| comp - same | −0.210 | 38.000 | 39.389 | 0.000 | 0.834 | 1.000 | 1.000 |
| inco - new | 2.360 | 34.471 | 18.667 | 0.475 | 0.018 | 0.110 | 0.073 |
| inco - same | −0.769 | 34.471 | 39.389 | 0.150 | 0.442 | 1.000 | 1.000 |
| new - same | −3.135 | 18.667 | 39.389 | 0.681 | 0.002 | 0.010 | 0.010 |

## C. Statistical analysis - $\Delta$ RT in novel versus learned transitions

**Supplementary Table 25. Output of the normality tests (Shapiro-Wilk test) of the $\Delta$ RT for the novel and learned transitions in each group in session 2 (start and end of session 2).** The analysis was performed in Python using the scipy package [2].

| | timepoint | condition | group | Shapiro-Wilk test statistic | p-value |
|---|---|---|---|---|---|
| 0 | session 2 start | learned | comp | 0.961135 | 0.652905 |
| 1 | session 2 end | learned | comp | 0.923847 | 0.194481 |
| 2 | session 2 start | learned | inco | 0.980506 | 0.967483 |
| 3 | session 2 end | learned | inco | 0.910030 | 0.116502 |
| 6 | session 2 start | learned | same | 0.945784 | 0.393335 |
| 7 | session 2 end | learned | same | 0.962028 | 0.669853 |
| 8 | session 2 start | novel | comp | 0.876945 | 0.028381 |
| 9 | session 2 end | novel | comp | 0.946798 | 0.440718 |
| 10 | session 2 start | novel | inco | 0.899585 | 0.079150 |
| 11 | session 2 end | novel | inco | 0.930404 | 0.221051 |
| 12 | session 2 start | novel | new | 0.954119 | 0.591482 |
| 13 | session 2 end | novel | new | 0.966819 | 0.808481 |

Since the $\Delta$ RT for novel transitions in the Compatible group showed a non-normal distribution at the start of session 2, we also report the output of non-parametric alternative statistical tests in the main article file.

## SUPPLEMENTARY REFERENCES


1. JASP Team, "JASP (Version 0.19.0)[Computer software]," (2024).
2. P. Virtanen, R. Gommers, T. E. Oliphant, *et al.*, "SciPy 1.0: fundamental algorithms for scientific computing in Python," Nat. Methods **17**, 261–272 (2020).
